# Morphology and phylogeny of ascomycetes associated with walnut trees (*Juglans regia*) in Sichuan province, China

**DOI:** 10.3389/fmicb.2022.1016548

**Published:** 2022-10-20

**Authors:** Xiu-Lan Xu, Fei-Hu Wang, Chao Liu, Han-Bo Yang, Zhen Zeng, Bao-Xin Wang, Ying-Gao Liu, Chun-Lin Yang

**Affiliations:** ^1^National Forestry and Grassland Administration Key Laboratory of Forest Resources Conservation and Ecological Safety on the Upper Reaches of the Yangtze River and Forestry Ecological Engineering in the Upper Reaches of the Yangtze River Key Laboratory of Sichuan Province, College of Forestry, Sichuan Agricultural University, Chengdu, Sichuan, China; ^2^Forestry Research Institute, Chengdu Academy of Agricultural and Forestry Sciences, Chengdu, Sichuan, China

**Keywords:** Dothideomycetes, fungal diversity, *Juglans regia*, phylogeny, Sordariomycetes, taxonomy

## Abstract

In Sichuan province, walnuts, consisting of *Juglans regia*, *Juglans sigillata*, and the hybrid *J. regia* × *J. sigillata*, are commercially important edible nuts, and *J. regia* is the most widespread plant. To date, the diversity and distribution of fungi inhabiting on *Juglans* have not received enough attention, although there have been studies focusing on pathogens from fruit and stem. In order to update the checklist of fungi associated with Sichuan walnuts, a survey on fungi associated with the three *Juglans* species from 15 representative regions in Sichuan was conducted. In this article, ten fungi distributed in two classes of Ascomycota (Dothideomycetes and Sordariomycetes) were described based on morpho-molecular analyses, and two novel species, *Neofusicoccum sichuanense* and *Sphaerulina juglandina*, a known species of *Ophiognomonia leptostyla*, and seven new hosts or geographical records of *Cladosporium tenuissimum*, *Diatrypella vulgaris*, *Helminthosporium juglandinum*, *Helminthosporium velutinum*, *Loculosulcatispora hongheensis*, *Periconia byssoides*, and *Rhytidhysteron subrufulum* were included. Morphological descriptions and illustrations of these fungi are provided.

## Introduction

The phylum Ascomycota contains the majority of described fungi and has 92,724 species (Kirk, 2022). The class Dothideomycetes, previously known as Loculoascomycetes, is the largest and most ecologically diverse class of Ascomycota (Barr, 1979a,b; Barr and Huhndorf, 2001; Hyde et al., 2013; Hongsanan et al., 2020a). It consists of 31,033 species from 36 orders and one order *Incertae sedis* (Kirk, 2022). Members of Dothideomycetes are mostly characterized by ascolocular ascoma development and bitunicate and fissitunicate asci (Barr and Huhndorf, 2001; Hyde et al., 2013). Sordariomycetes is the second largest class, comprising 23,187 species classified into 47 orders and one order *Incertae sedis* (Kirk, 2022), and it is characterized by non-lichenized, flask-shaped, less frequently cleistothecial ascomata and unitunicate asci (Maharachchikumbura et al., 2016; Hyde et al., 2020). Both the two classes have a cosmopolitan distribution and can be found in almost all ecosystems including terrestrial, marine, and freshwater habitats (Luo et al., 2019; Dong et al., 2020; Jones et al., 2020). Some taxa are pathogenic to plants, arthropods, nematodes and mammals (Spatafora et al., 2015; Chang et al., 2019; Norphanphoun et al., 2019; Hongsanan et al., 2020a; Hyde et al., 2020; Xu et al., 2021), and fungicolous (Sun et al., 2019; Yang et al., 2019). Some members act as endophytes and saprobes (Jeewon et al., 2017; Rashmi et al., 2019; Mortimer et al., 2021; Xu et al., 2022). As the outline of Dothideomycetes and Sordariomycetes has been frequently revised (Hyde et al., 2013, 2020; Wijayawardene et al., 2014; Maharachchikumbura et al., 2015, 2016; Hongsanan et al., 2020a,b), numerous novel species, genera, families, and orders have been discovered (Maharachchikumbura et al., 2021; Pem et al., 2021; Zeng et al., 2022). The taxonomy of the two classes remains in a perpetual transitional state. However, further research will be carried out to promote the modern classification of these two classes, incorporating morphology and phylogenies (Lumbsch and Huhndorf, 2007, 2010; Maharachchikumbura et al., 2021) and following the “one fungus-one name” concept (Taylor, 2011).

China is leading the way in discovery of new fungal species and is where the highest fungal species were discovered than any other countries in 2020 (Wang et al., 2021; Bhunjun et al., 2022). In recent years, there has been increasing attention on species and distribution of fungi in China. More studies concerning fungal diversity, especially in Dothideomycetes and Sordariomycetes, were reported (Raza et al., 2019; Zhang and Cai, 2019). For instance, the saprophytic fungi of wood were investigated in some regions of China by Wang (2014). Referring to the checklist of pathogenic fungi on *Citrus* and apple trees, pathogens from Dothideomycetes and Sordariomycetes are the main casual agents of fungal disease (Dai et al., 2021; Zheng et al., 2022). Raza et al. (2019) described diverse new genus, new species, and new records of pathogenic fungi associated with sugarcane in southern China and belonging to the two classes. According to a diversity investigation of bambusicolous ascomycetes from Yunnan, China, most species belong to classes Dothideomycetes (37.01%) and Sordariomycetes (60.85%) (Dai et al., 2022). To date, compared with other regions in China, viz. Yunnan, and Guizhou, where fungal diversity and new taxa have been documented (Bao et al., 2018; Luo et al., 2018; Feng et al., 2019, 2021; Chen et al., 2020; Mortimer et al., 2021; Wijayawardene et al., 2021; Dai et al., 2022), there is a lack of monographs on fungi associated with specific plant substrates in Sichuan province. Furthermore, there are few scientific evaluations conducted for fungi in national nature reserves, national forest parks, and other regions or hosts in Sichuan province, China.

The walnut family (Juglandaceae) is mainly distributed in North America, Europe, and East Asia, and consists of eight genera and more than 60 species (Jahanban-Esfahlan et al., 2019; Guo et al., 2020; Zhang et al., 2022). These trees are important resources for timber, furniture, nuts, and cultivated ornamentals. The most well-known genera of Juglandaceae are *Juglans* and *Carya*, in which 22 species and 1 variety, and 18 species, 4 varieties and 9 hybrids are included, respectively, following The Plant List (2022). China is the leading world producer of walnuts, followed by France, India, Iran, Romania, Turkey, Ukraine, and the United States (Martínez et al., 2010; Fan et al., 2018), where five walnut species, the Persian walnut (*Juglans regia* L.), Iron walnut (*Juglans sigillata* Dode), Manchurian walnut (*J. mandshurica* Maxim.), Ma walnut (*J. hopeiensis* Hu), and Chinese hickory (*C. cathayensis* Sarg.) are the major native species, and then the eastern American black walnut (*J. nigra* L.), Arizona walnut [*J. major* (Torr.) A. Heller], Texas walnut (*J. macrocarpa* Texas), and Pecans [*C. illinoinensis* (Wangenh.) K. Koch] were introduced (Xi, 1987; Pei and Lu, 2011). At present, the walnut species *J. regia*, *J. sigillata*, and *C. cathayensis* are widely cultivated as economic fruit trees in diverse regions of China (Xi, 1987; Ma et al., 2019). In addition, the species *J. regia* is the common walnut and is broadly cultivated because of the commercial high-added value of the seeds (Jahanban-Esfahlan et al., 2019). Sichuan province is located at the boundary of the natural distribution of northern (north of the Nibashan Qinling Mountains) and southern (south of the Erlangshan Nibashan-Huangmaogeng-Wumengshan Mountains) walnuts in China. Generally, the northern walnut is classified as *J. regia*, while the southern walnut is classified as *J. sigillata*. The Sichuan walnut is mainly composed of *J. regia*, *J. sigillata*, and the hybrid *J. regia* × *J. sigillata* by FISH, early-fruiting gene analysis, and SSR analysis (Luo and Chen, 2020), and among them *J. regia* has the most extensive distribution range.

A review of the literature and records from the United States National Fungus Collections (Farr and Rossman, 2022) on *Juglans*-associated fungi reveals that nearly 280 species, which belong to 134 genera of Ascomycetes, have so far been described or recorded worldwide. Most of the species belong to classes Dothideomycetes (35.4%), Sordariomycetes (48.2%), and Leotiomycetes (8.9%). Among them, Botryosphaeriales, Pleosporales, Diaporthales, Hypocreales, Xylariales, and Helotiales fungi are the most diverse on walnut trees and account for 39.4 and 37.4% in Dothideomycetes, 35.6, 23, and 18.5% in Sordariomycetes, and 96% in Leotiomycetes. Furthermore, there are 213 (98 genera), 63 (44 genera), 32 (27 genera), and 26 (23 genera) species associated with *J. regia*, *J. nigra*, *J. cinerea*, and *J. mandshurica*, respectively. Moreover, a review of about 350 bodies of literature on walnut pathogenic fungi, which are mainly reported in China, the United States, Iran, and Italy in 1930–2022, reveals that nearly 115 species of Ascomycetes belonging to Dothideomycetes (24.3%), Sordariomycetes (69.6%), and Leotiomycetes (6.1%) have been described or recorded. Botryosphaeriaceae and Diaporthaceae fungi have been described as the main causal agents of branch dieback and shoot blight of walnut trees (*Juglans* species), including the *Botryosphaeria*, *Diplodia*, *Dothiorella*, *Lasiodiplodia*, *Neofusicoccum* species in Botryosphaeriaceae, and *Diaporthe* in Diaporthaceae (Chen et al., 2014; Dissanayake et al., 2017; Fan et al., 2018; Agustí-Brisach et al., 2019; Eichmeier et al., 2020; Jiménez-Luna et al., 2020; López-Moral et al., 2020, 2022). In addition, the *Colletotrichum*, *Fusarium*, and *Phaeoacremonium* species are also recorded as the pathogen of walnut fruit necrosis, twig canker, and leaf spot (Montecchio et al., 2015; Savian et al., 2019; Sohrabi et al., 2020; Han et al., 2021).

According to literature, there are few thorough studies on the taxonomy and phylogeny of fungi on walnut trees. Beginning in 2020, a special investigation was conducted in the main walnut planting areas across Sichuan province involving 15 regions of eight cities or autonomous prefecture. In this study, we aim to describe new findings and contribute to fungal diversity on walnut trees by focusing on new species, new records, and new sexual-asexual connections. Fungal specimens in various tissues of the host plant were collected and examined. A multigene phylogeny integrated with morphological comparison was carried out to determine the classification of the new collections.

## Materials and methods

### Specimen collection and morphological study

Specimens of twigs and leaves from walnut trees were collected and taken back to the laboratory in a sampling bag during the investigation in Sichuan province, China. The host, locality, and time were documented in the field. The substrate with fruiting bodies was checked following the methods described in Senanayake et al. (2020). Single ascospore or single conidium isolations were carried out following the method described by Chomnunti et al. (2014). Germinating spores were transferred to PDA, incubated at 25°C with a 12-h photoperiod. After incubation for 7 days to 2 months depending on growth rate, colonies were examined for their diameter, shape, and appearance. Ascomata and conidiomata were observed and photographed using a dissecting microscope, NVT-GG (Shanghai Advanced Photoelectric Technology Co., Ltd., Shanghai, China) fitted with a VS-800C micro-digital camera (Shenzhen Weishen Times Technology Co., Ltd., Shenzhen, China). Dimensions of asexual and sexual structures, viz. ascomata, peridia, asci, ascospores, conidiophores, conidiogenous cells, paraphyses, conidiomata wall, and conidia, and numbers of septa were based on field samples and were photographed using an Olympus BX43 compound microscope fitted with an Olympus DP22 digital camera in association with the ACDSee (v3.1) software, and an Olympus BX53 compound microscope fitted with an SD1600AC digital camera in association with the CapStudio (3.8.10.0) software (Image Technology company, Suzhou, China). Measurements were conducted using Tarosoft^®^ Image Frame Work v.0.9.7 [Tarosoft (R) Nonthaburi, Thailand]. A lactophenol cotton blue reagent was used to observe the number of septa. The iodine reaction of the ascus wall was tested in Melzer’s reagent (MLZ). Type specimens were deposited at the Herbarium of Sichuan Agricultural University, Chengdu, China (SICAU). Ex-type cultures were deposited at the Culture Collection in Sichuan Agricultural University (SICAUCC), and MycoBank numbers were registered.^1^

### DNA extraction, amplification, and sequencing

For each isolate, total genomic DNA was extracted from mycelia that were grown on PDA at 25°C for 2 weeks using Plant Genomic DNA Extraction Kit (Tiangen, China). Internal transcribed spacer regions (ITS), partial large subunit nuclear rDNA regions (LSU), and partial small subunit nuclear rDNA regions (SSU) were amplified with primer pairs ITS5/ITS4 (White et al., 1990), LR0R/LR5 (Vilgalys and Hester, 1990), and NS1/NS4 (White et al., 1990), and translation elongation factor 1-alpha gene (*tef*1-a) was amplified with primer pairs EF1-983F/EF1-2218R (Rehner and Buckley, 2005), EF1-728F/EF1-986R (Carbone and Kohn, 1999), EF1-1567R (Stielow et al., 2015), and EF2 (O’Donnell et al., 1998). The RNA polymerase II second largest subunit (*rpb*2) was amplified with primer pairs fRPB2-5F/fRPB2-7cR (Liu et al., 1999), and primer pairs T1/T22 (O’Donnell and Cigelnik, 1997), and β-Sandy-R (Stukenbrock et al., 2012) were used for the β-tubulin gene (*tub*2). Primer pair MS204 E1F1/MS204 E5R1a (Walker et al., 2012a) was for the guanine nucleotide-binding protein subunit beta gene (*ms*204), and ACT-512F/ACT-783R (Carbone and Kohn, 1999) was for the actin gene (*act*).

Polymerase chain reaction (PCR) was performed in a 25-μl reaction mixture containing 22 μl Master Mix (Beijing TsingKe Biotech Co., Ltd., Beijing, China), 1 μl DNA template, and 1 μl of each primer (10 μM). The PCR thermal cycle program for LSU, SSU, ITS, *tef*1-α, and *rpb*2 genes was amplified following Dai et al. (2016) and Xu et al. (2020). The amplification reactions for the *ms*204, *tub*2, and *act* genes were performed as described by Videira et al. (2016) and Gong et al. (2017). PCR products were sequenced at TsingKe Biological Technology Co., Ltd. (Chengdu, China). The newly generated sequences were deposited on GenBank.

### Sequence alignment and phylogenetic analyses

Phylogenetic analyses were conducted based on the combined dataset. Raw sequences were edited and assembled with BioEdit version 7.0.5.3 (Hall, 1999). The assembled sequences were used to query for nucleotide sequences by BLAST with the default settings on the NCBI web server.^2^ Highly similar sequences derived from reliable publications are downloaded and listed in Supplementary Table 1. Alignments were performed using the MAFFT v.7.429 online service (Katoh et al., 2019), and ambiguous regions were excluded using BioEdit version 7.0.5.3. Multigene sequences were concatenated with the Mesquite software (Maddison and Maddison, 2019) for further phylogenetic analyses. Phylogenetic trees were inferred with maximum likelihood (ML) and Bayesian analyses (BI) according to details in Xu et al. (2022).

### Pathogenicity test

After species identification, healthy 3-year-old walnut seedlings and 10-year-old walnut trees of *J. regia* were inoculated with five fungal species isolated from symptomatic plants, viz. *Cladosporium tenuissimum*, *Neofusicoccum sichuanense*, *Ophiognomonia leptostyla*, *Periconia byssoides*, and *Sphaerulina juglandinum*, to determine their pathogenicity. The strains were cultured for a duration of 5–30 days at room temperature (25°C) on PDA with a 12-h fluorescent light/dark regime. Conidial inoculation was conducted for three strains (*Sp. juglandinum*: SICAUCC 22-0108; *O. leptostyla*: SICAUCC 22-0103; *Cl. Tenuissimum*: SICAUCC 22-0110). Conidial suspensions (10^5^ conidia/ml) were sprayed onto wound sites created with a sterile inoculation needle (Desai et al., 2019). Mycelial inoculation was conducted for strains of *N. sichuanense* (SICAUCC 22-0094) and *P. byssoides* (SICAUCC 22-0107). A PDA mycelial plug (5-mm diameter) was put on each wound that was produced with a sterile inoculation needle on leaves or a wood cutting knife on stem. Each wound was coated with moistened cotton wool and covered with parafilm. The parafilm and cotton wool were removed after 3 days. Twenty inoculation dots were set for each isolate, twenty replicates were sprayed with sterile water and uncolonized PDA plugs served as controls. The inoculated plants were maintained in the field to observe for symptoms. Re-isolation from the infected tissue on PDA and morphological observation of fungal pathogens were performed to verify Koch’s postulates.

## Results

### Phylogenetic analyses

Based on the results of BLAST on GenBank, our isolates belong to 9 genera: *Cladosporium*, *Diatrypella*, *Helminthosporium*, *Sulcatisporaceae*, *Neofusicoccum*, *Ophiognomonia*, *Periconia*, *Rhytidhysteron*, and *Sphaerulina*. To infer the relationship of our isolates in the genera, 9 phylogenetic analyses were performed in this study.

#### *Cladosporium cladosporioides* species complex phylogeny

A combined ITS, *tef*1-a, and *act* sequences dataset, including 90 in-group taxa and two out-group taxa (*Cl. longissimum*, CBS 300.96; *Cl. sphaerospermum*, CBS 193.54), was used to construct the phylogenetic tree (Supplementary Figure 1). The alignment contained 1,851 characters including gaps. The best scoring randomized axelerated maximum likelihood (RAxML) tree with a final likelihood value of −16,923.095557 is presented. The matrix had 921 distinct alignment patterns, with 37.63% of undetermined characters or gaps. Estimated base frequencies were as follows: A = 0.231786, C = 0.293167, G = 0.248251, and T = 0.226797, with substitution rates AC = 1.872991, AG = 3.80827, AT = 1.958762, CG = 1.140769, CT = 6.33381, and GT = 1. The gamma distribution shape parameter α = 0.220947 and the tree length = 4.39734. The Bayesian analysis resulted in 160,002 trees after 8,000,000 generations, from which 120,002 were used for calculating posterior probabilities after the first 25% of trees representing the burn-in phase were discarded.

#### *Diatrypella* species phylogeny

A combined ITS and *tub*2 dataset was used for phylogenetic analyses (Supplementary Figure 2). The alignment contained 37 isolates, and the tree was rooted to *Neoeutypella baoshanensi* (GMB0052, LC 12111). The final alignment contained a total of 2,257 characters used for the phylogenetic analyses and included gaps. The best scoring RAxML tree with a final likelihood value of −7,996.010848 is presented. The matrix had 726 distinct alignment patterns, with 49.81% of undetermined characters or gaps. Estimated base frequencies were as follows: A = 0.219422, C = 0.275801, G = 0.23297, and T = 0.271808, with substitution rates AC = 0.780583, AG = 3.041274, AT = 1.166226, CG = 0.884206, CT = 4.451043, and GT = 1. The gamma distribution shape parameter α = 0.318927, and the tree length = 0.838444. The Bayesian analysis resulted in 3,502 trees after 5,000,000 generations, from which 2,628 were used for calculating posterior probabilities after the first 25% of trees representing the burn-in phase were discarded.

#### *Helminthosporium* species phylogeny

A combined ITS-LSU-SSU-*rpb*2-*tef*1-α dataset comprising 25 taxa in *Helminthosporium* and one out-group taxon (*Massarina cisti*, CBS 266.62) was used for phylogenetic analyses (Supplementary Figure 3). The alignment contained 4,602 characters including gaps. The best scoring RAxML tree with a final likelihood value of −17,311.827379 is presented. The matrix had 1,117 distinct alignment patterns, with 32.07% of undetermined characters or gaps. Estimated base frequencies were as follows: A = 0.238622, C = 0.253941, G = 0.269616, and T = 0.237821, with substitution rates AC = 1.964004, AG = 5.639629, AT = 1.986722, CG = 1.097192, CT = 10.702449, and GT = 1. The gamma distribution shape parameter α = 0.125261, and the tree length = 1.171688. The Bayesian analysis resulted in 17,802 trees after 8,000,000 generations, from which 13,352 were used for calculating posterior probabilities after the first 25% of trees representing the burn-in phase were discarded.

#### *Sulcatisporaceae* species phylogeny

For phylogenetic analyses, we used a combined dataset (ITS, LSU, SSU, *tef*1-α, and *rpb*2) comprising 13 taxa from 9 genera within the family and 2 out-group taxa (*Leucaenicola phraeana* MFLUCC 18-0472, *L. aseptata* MFLUCC 17-2423) in Bambusicolaceae (Supplementary Figure 4). The alignment contained 5,264 characters including gaps. The best scoring RAxML tree with a final likelihood value of −18,207.015800 is presented. The matrix had 1,271 distinct alignment patterns, with 26.59% of undetermined characters or gaps. Estimated base frequencies were as follows: A = 0.241466, C = 0.255136, G = 0.269143, and T = 0.234255, with substitution rates AC = 1.094159, AG = 2.768055, AT = 1.059894, CG = 0.695463, CT = 5.978163, and GT = 1. The gamma distribution shape parameter α = 0.140781, and the tree length = 0.919701. The Bayesian analysis resulted in 1,102 trees after 1,000,000 generations, from which 828 were used for calculating posterior probabilities after the first 25% of trees representing the burn-in phase were discarded.

#### *Neofusicoccum* species phylogeny

For phylogenetic analyses, we used a combined dataset (ITS, *rpb*2, *tef*1-α, and *tub*2) comprising 63 taxa and including 128 isolates in *Neofusicoccum* and one out-group taxon (*Botryosphaeria dothidea*, CBS 100564) (Supplementary Figure 5). The alignment contained 2,272 characters including gaps. The best scoring RAxML tree with a final likelihood value of −9,986.846540 is presented. The matrix had 870 distinct alignment patterns, with 27.58% of undetermined characters or gaps. Estimated base frequencies were as follows: A = 0.217491, C = 0.292821, G = 0.272990, and T = 0.216697, with substitution rates AC = 1.078167, AG = 4.928923, AT = 1.167073, CG = 1.17778, CT = 9.092502, and GT = 1. The gamma distribution shape parameter α = 0.25616, and the tree length = 0.895474. The Bayesian analysis resulted in 40,502 trees after 50,000,000 generations, from which 30,378 were used for calculating posterior probabilities after the first 25% of trees representing the burn-in phase were discarded.

#### *Ophiognomonia* species phylogeny

For phylogenetic analyses, we used a combined dataset (ITS, *ms*204, and *tef*1-α) comprising 49 taxa and including 62 isolates in *Ophiognomonia* and one out-group taxon (*Ambarignomonia petiolorum*, CBS 121227) (Supplementary Figure 6). The alignment contained 3,019 characters including gaps. The best scoring RAxML tree with a final likelihood value of −28,800.079248 is presented. The matrix had 1,644 distinct alignment patterns, with 22.23% of undetermined characters or gaps. Estimated base frequencies were as follows: A = 0.227917, C = 0.301009, G = 0.23455, and T = 0.236524, with substitution rates AC = 1.22843, AG = 2.406365, AT = 1.296931, CG = 0.947939, CT = 4.032567, and GT = 1. The gamma distribution shape parameter α = 0.362898, and the tree length = 3.168442. The Bayesian analysis resulted in 29,902 trees after 8,000,000 generations, from which 22,428 were used for calculating posterior probabilities after the first 25% of trees representing the burn-in phase were discarded.

#### *Periconia* species phylogeny

For phylogenetic analyses, we used a combined dataset (ITS, LSU, SSU, and *tef*1-α) comprising 70 isolates from Didymosphaeriaceae, Lentitheciaceae, Massarinaceae, Morosphaeriaceae, and Periconiaceae (Supplementary Figure 7). The tree is rooted to *Morosphaeria ramunculicola* (NBRC 107813) and *M. velatispora* (NBRC 107812). The alignment contained 4,562 characters including gaps. The best scoring RAxML tree with a final likelihood value of −21,102.547135 is presented. The matrix had 1,342 distinct alignment patterns, with 41.61% of undetermined characters or gaps. Estimated base frequencies were as follows: A = 0.240683, C = 0.251101, G = 0.26776, and T = 0.240456, with substitution rates AC = 1.392883, AG = 2.46189, AT = 1.701375, CG = 1.147404, CT = 7.975303, and GT = 1. The gamma distribution shape parameter α = 0.2038, and the tree length = 1.571973. The Bayesian analysis resulted in 36,502 trees after 8,000,000 generations, from which 27,378 were used for calculating posterior probabilities after the first 25% of trees representing the burn-in phase were discarded.

#### *Rhytidhysteron* species phylogeny

For phylogenetic analyses, we used a combined dataset (ITS, LSU, SSU, and *tef*1-α) comprising 21 taxa and including 50 isolates in *Rhytidhysteron* and two out-group taxa of *Hysterographium fraxini* (MFLU 15-3681, CBS 109.43) (Supplementary Figure 8). The alignment contained 3,652 characters including gaps. The best scoring RAxML tree with a final likelihood value of −11868.374118 is presented. The matrix had 918 distinct alignment patterns, with 22.28% of undetermined characters or gaps. Estimated base frequencies were as follows: A = 0.24053, C = 0.249365, G = 0.274418, and T = 0.235688, with substitution rates AC = 1.370478, AG = 2.713904, AT = 1.18812, CG = 1.104666, CT = 6.269731, and GT = 1. The gamma distribution shape parameter α = 0.156243, and the tree length = 0.575373. The Bayesian analysis resulted in 11,402 trees after 8,000,000 generations, from which 8,552 were used for calculating posterior probabilities after the first 25% of trees representing the burn-in phase were discarded.

#### *Sphaerulina* species phylogeny

A combined ITS-LSU-*rpb*2-*tef*1-α-*tub*2 dataset was used for phylogenetic analyses (Supplementary Figure 9). The alignment contained 64 isolates, and the tree was rooted to *Septoria scabiosicola* (CBS 102334 and CBS 108981). The alignment contained 3,454 characters including gaps. The best scoring RAxML tree with a final likelihood value of −18735.843276 is presented. The matrix had 1,278 distinct alignment patterns, with 34.04% of undetermined characters or gaps. Estimated base frequencies were as follows: A = 0.241431, C = 0.252483, G = 0.283959, and T = 0.222127, with substitution rates AC = 1.488577, AG = 3.234311, AT = 1.027066, CG = 0.922236, CT = 6.45922, and GT = 1. The gamma distribution shape parameter α = 0.179629, and the tree length = 2.009249. The Bayesian analysis resulted in 20,002 trees after 1,000,000 generations, from which 15,002 were used for calculating posterior probabilities after the first 25% of trees representing the burn-in phase were discarded.

All the phylogenetic trees for the above taxa resulting from the Bayesian analysis had a topology identical to the ML tree presented. In both analyses (ML and Bayesian), the phylogenetic status of our isolates was resolved in a well-supported clade. The phylogenetic results obtained for each dataset are discussed where applicable in the descriptive notes below.

### Taxonomy

In the present study, 20 specimens have been collected from *J. regia*. Ten fungal species in Dothideomycetes and Sordariomycetes were isolated, and eight strains belonging to two new species were found. These taxa are subsequently described below.

#### *Cladosporium tenuissimum* Cooke, Grevillea 6: 140 (1878)

**FIGURE 1:**
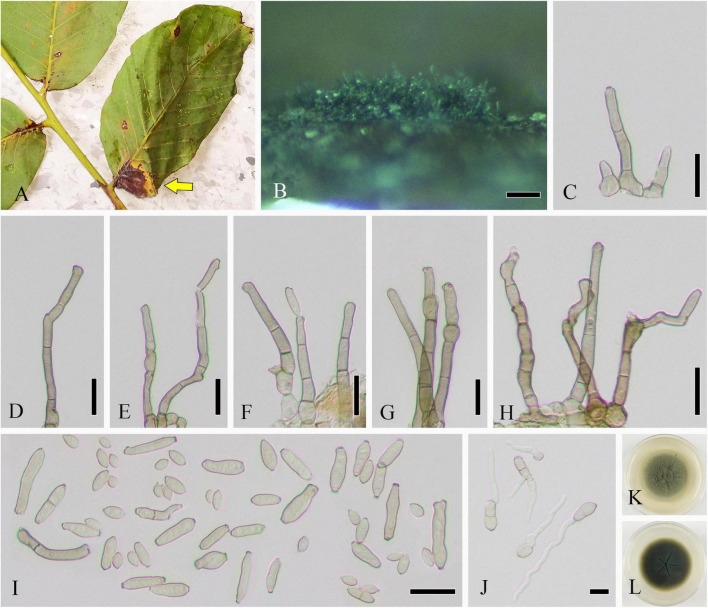
*Cladosporium tenuissimum* (SICAU 22-0109). **(A)** Symptoms of necrotic lesions observed on host. **(B)** Appearance of colonies on substrate. **(C–H)** Conidiophores. **(I)** Ramoconidia and conidia. **(J)** Germinating ramoconidia and conidia. **(K,L)** Colonies on PDA for 10 days. Scale bars: **(B)** 100 μm and **(C–J)** 20 μm.

Parasitic on living leaves of *J. regia* causing necrosis. **Sexual morph:** Undetermined. **Asexual morph (Figure 1):** Hyphomycetous. *Mycelia* sparse, mainly immerse, and often forming necrotic lesions with abundant sporulation on the reverse. *Conidiophores* 19–120 × 3–9 μm ($\bar{x}$ = 73 × 6 μm, *n* = 50), solitary or in groups, fasciculate, micronematous, macronematous, arising terminally and laterally from swollen hyphae, erect, straight or flexuous, cylindrical-oblong, slightly attenuated toward the apex, subnodulose or nodulose, occasionally branched, 1–7-septate, sometimes distinctly constricted at septa, olivaceous or olivaceous brown, more or less thickened walled, and pronounced loci crowded at the apex. Micronematous conidiophores reduced to conidiogenous cells. *Conidiogenous cells* 12–20 (–30) × 3–8 μm ($\bar{x}$ = 16 × 5 μm, *n* = 50), integrated, mainly terminal, cylindrical-oblong to subclavate, aseptate, sometimes uniseptate, sometimes geniculate, non-nodulose, polyblastic, with 1–3(–4) apically crowed loci, conspicuous, 0.5–3 μm diameter, and thickened and somewhat darkened conidiogenous loci. *Ramoconidia* 6–15 × 3–8 μm ($\bar{x}$ = 10 × 5 μm, *n* = 50), cylindrical-oblong, 0(–1)-septate, pale olivaceous, smooth, and base truncate. *Conidia* numerous, catenate, forming short branched chains in all directions, and aseptate. *Small terminal conidia* 4–9 × 3–7 μm ($\bar{x}$ = 6 × 4 μm, *n* = 50), globose, subglobose or ovoid to obovoid, and apex rounded or somewhat attenuated. *Intercalary conidia* 6–10 × 3–5 μm ($\bar{x}$ = 8 × 4 μm, *n* = 20), limoniform to ellipsoid, aseptate, rarely uniseptate, and with 1–2 distal hila. *Secondary ramoconidia* sparsely forming. *Hila* 0.5–1.5 μm, and conspicuous diameter. *Microcyclic conidiogenesis* not observed.

**Culture characteristics:** Colonies on PDA attaining 30–45 mm diameter in 1 week, gray, powdery to fluffy, sporulation profuse, radially furrowed, without prominent exudates, reverse olivaceous, pale green to grayish white toward margins, and deep radial fissures. Colonies on MEA reaching 40–50 mm diameter after 1 week, grayish white to gray, colony wrinkled and folded, radially furrowed, sporulation profuse, without prominent exudates, reverse olivaceous to olivaceous brown, margin grayish white, and radial fissures. Colonies on OA reaching 20–30 mm diameter after 1 week, grayish olivaceous with profuse spores, reverse olivaceous to olivaceous brown, and without exudates. Colonies on SNA reaching 15–20 mm after 7 days, pale olivaceous, aerial mycelium scanty, loose, sporulation medium, reverse olivaceous, and without exudates.

**Habitat and host range:** Different host plants isolated from dead leaves, twigs, stems, wood and other organic matter; also isolated from air, bread, soil, and water.

**Distribution:** Cosmopolitan but especially common in the tropics.

**Material examined:** China, Sichuan province, Guangyuan city, 106°10′43.81″E, 32°12′30.59″N, alt. 485 m, on leaves of *J. regia*, 11 July 2020, H. B. Yang and C. L. Yang, YHB202007003 (SICAU 22-0109), living culture SICAUCC 22-0110.

**Note:** Given the overlapping of the key differential features on morphology, species identification of *Cladosporium* is currently based on multi-locus analysis. The preliminary phylogeny based on a concatenated ITS-*act*-*tef*1-α sequence matrix of representative strains in *Cladosporium* indicates that our isolate belongs to the *Cladosporium cladosporioides* species complex. The phylograms from ML and BI analyses were similar in overall topologies. This isolate is phylogenetically grouped with the epitype of *Cl. tenuissimum* (CBS 125995) with strong statistical support (94% ML, 0.94 PP) (Supplementary Figure 1). The nucleotide comparison of ITS, *act*, and *tef*1-α showed 100% (540/540; 0 gaps), 97.7% (215/220; 0 gaps), and 99.7% (436/437; 0 gaps) identities with isolate CBS 125995. Consistent with the common points of *Cl. tenuissimum*, the conidiophores of our isolate often possess a slightly swollen head-like apex and sometimes have intercalary subnodulose or nodulose swellings, being quite apart from the apical cell. Furthermore, the morphological comparison shows great differences in size among various collections, especially in conidiophores, conidiogenous cells, and ramoconidia (Bensch et al., 2010, 2012).

#### *Diatrypella vulgaris* Trouillas, W. M. Pitt and Gubler, Fungal Diversity 49: 212 (2011)

**FIGURE 2:**
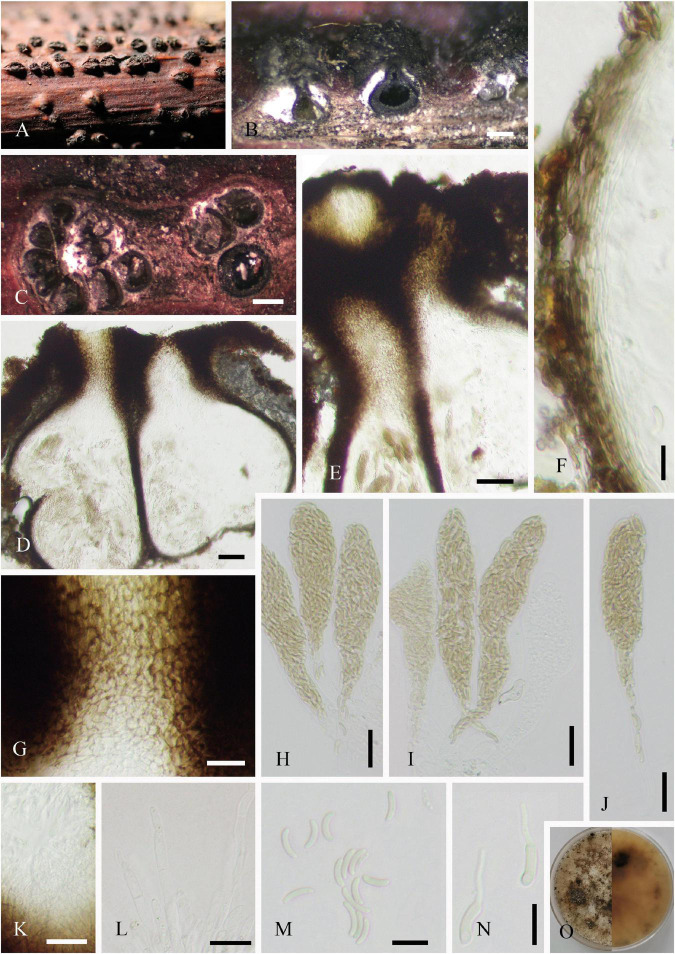
*Diatrypella vulgaris* (SICAU 22-0101). **(A)** Stromata on host substrate. **(B)** Vertical section through stromata. **(C)** Transverse sections through stromata. **(D)** Section through the locules. **(E,G,K)** Ostiolar canal. **(F)** Peridium. **(H–J)** Asci. **(L)** Paraphyses. **(M)** Ascospores. **(N)** Germinating ascospores. **(O)** Colony on PDA for 15 days. Scale bars: **(B,C)** 200 um, **(D,E)** 50 um, **(F)** 10 um, **(G–L)** 20 um, and **(M,N)** 10 um.

Saprobic on dead twigs of *J. regia*. **Sexual morph (Figure 2):** *Stromata* well developed, scattered, 250–902 × 281–1,006 μm ($\bar{x}$ = 536 × 604 μm, *n* = 20), pustulate, black, rounded to irregular in shape on host surface, semi-immersed, erumpent through host bark, and with 3–9 locules immersed in single stroma. *Endostroma* consists of outer dark brown, small, dense, thin parenchymal cells, and an inner layer of white, large, loose parenchymal cells. *Ostiole* opening separately, papillate or apapillate, central 162–353 μm high, 140–205 μm diameter ($\bar{x}$ = 231 × 177 μm, *n* = 10). *Locules* immersed in stroma, circular to ovoid, with cylindrical neck, brevicollous or longicollous, 223–601 μm high, and 200–398 μm diameter ($\bar{x}$ = 370 × 274 μm, *n* = 20). *Peridium* composed of outer layer of dark brown to black, thin-walled cells, arranged in *textura angularis* to *textura prismatica*, and inner layer of hyaline thin-walled cells of *textura prismatica*. *Paraphyses* 3–7 μm wide ($\bar{x}$ = 5 μm, *n* = 30), elongate cylindrically, septate, branched, and tapering toward the apex. *Asci* 115–158 × 18–28.5 μm ($\bar{x}$ = 141 × 23 μm, *n* = 20), unitunicate, polysporous, clavate, with thin-walled pedicel, apically rounded, and J^–^ in Melzer’s reagent. *Ascospores* 6–12 × 1–3 μm ($\bar{x}$ = 9 × 2 μm, *n* = 20), overlapping, crowded, allantoid, slightly or moderately curved, smooth, subhyaline, yellowish in mass, and aseptate. **Asexual morph:** Undetermined.

**Culture characteristics:** Ascospores germinated on PDA within 12 h. Colonies on PDA reached 60 mm diameter after 1 week, circular, flat, white when young, became pale brown with age, dense, thinning toward edge, reverse side similar in color, formed black pycnidia after 14 days, and excluded conidia in light orange masses.

**Habitat and host range:** On decaying wood of *Citrus paradisi*, *Fraxinus angustifolia*, *Schinus molle* var. *areira*, and some unidentified plants.

**Distribution:** Australia, China, and Thailand.

**Material examined:** China, Sichuan province, Luding county, 102°11′34″E, 29°40′32″N, alt. 1,306 m, on twigs of *J. regia*, 18 July 2021, F. H. Wang, WFH202107011 (SICAU 22-0101), living culture SICAUCC 22-0102.

**Note:** This isolate resembles the species of *Diatrypella*, in pustule-like stromata erumpent through the host surface, polysporous asci, and allantoid ascospores. Morphologically, it shows the same features as *Diatrypella vulgaris*, but the asci of our isolate (SICAUCC 22-0102) are bigger than those of HVGRF03 (80–130 × 18–20 μm), MFLUCC 17-0128 (90–130 × 14–19 μm), and GMB0051 (111.4–152.9 × 10.6–17.5 μm). When compared with other isolates on the ascospores, it has similar dimension with isolates HVGRF03 and GMB0051, but bigger than MFLUCC 17-0128 (4.5–7.5 × 1–2 μm). Phylogenetically, SICAUCC 22-0102 is clustered together with HVFRF03 and HVFRA02 with strong bootstrap support (98% ML, Supplementary Figure 2). The comparisons of ITS and *tub*2 sequences in NCBI both showed 100% (ITS = 530/530; 0 gaps; *tub*2 = 358/358; 0 gaps) similarity to the strain of *D. vulgaris* (HVGRF03) from holotype specimens. *D. vulgaris* has been reported in Australia, Thailand, and China (Trouillas et al., 2011; Hyde et al., 2017; Long et al., 2021). In this study, we introduce *D. vulgaris* as a new record from Sichuan province in China, and this is the first report on the genus *Juglans*.

#### *Helminthosporium juglandinum* Voglmayr and Jaklitsch, Stud. Mycol. 87: 59 (2017)

**FIGURE 3:**
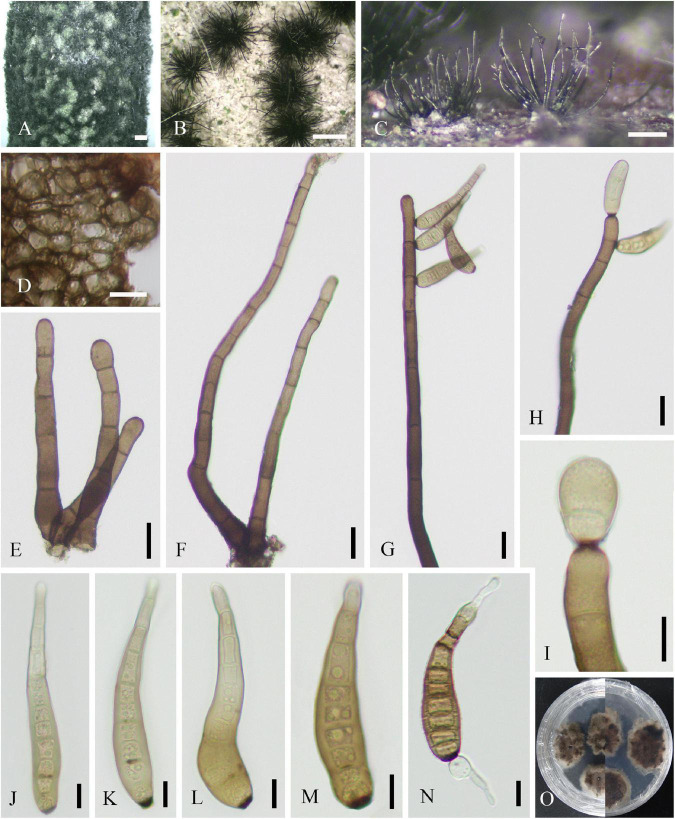
*Helminthosporium juglandinum* (SICAU 22-0090). **(A–C)** Colonies in face view. **(D)** Stroma cells in section. **(E,F)** Conidiophores. **(G–I)** Conidiophore apices with apical and lateral conidia. **(J–M)** Conidia. **(N)** Germinating conidium. **(O)** Colony on PDA for 20 days. Scale bars: **(A)** 1,000 um, **(B)** 500 um, **(C)** 200 um, **(D–H)** 20 um, and **(I–N)** 10 um.

Saprobic on dead twigs of *J. regia*. *Colonies* on natural substrate discrete, punctiform, sometimes confluent, usually in large groups, and black. **Sexual morph:** Undetermined. **Asexual morph (Figure 3):** *Mycelia* mostly immersed, and on the surface forming small stroma-like aggregations of red brown pseudoparenchymatous stromal cells (7–17 × 10–26, $\bar{x}$ = 12 × 16 μm, *n* = 20). *Conidiophores* fasciculate, arising from the upper cells of the stromata, erect, simple, straight or flexuous, thick-walled, sub-cylindrical, smooth, brown to dark brown, with a defined apical pore at the apex, measuring 127–585 μm long ($\bar{x}$ = 360 μm, *n* = 20), 15–20 μm wide ($\bar{x}$ = 17 μm, *n* = 20) at the base, and 7–13 μm wide ($\bar{x}$ = 10 μm, *n* = 20) near the slightly inflated apex. *Conidia* 63–99 × 11–15 (–21) μm ($\bar{x}$ = 79 × 13 μm, *n* = 20), tapering to 3–6 μm ($\bar{x}$ = 5 μm, *n* = 20) at the distal end, with a 4- to 7-μm wide ($\bar{x}$ = 5 μm, *n* = 20) blackish-brown scar at the base, rostrate, straight or flexuous, thick-walled, smooth, pale brown, and 4–11-distoseptate.

**Culture characteristics:** Conidia germinated on PDA within 12 h, and the germ tubes were produced from both ends. Cultures grew slowly on PDA, and colonies reached 2 cm in diameter after 20 days at 25°C. Colony was thin, dense, with irregular fimbriate edge, gray brown, and fruity. Mycelium radiated outward, hairy, sparse, dark, superficial, and partly immersed.

**Habitat and host range:** Dead corticated twigs of *J. regia*, fungicolous on conidiomata of *Diaporthe* sp.

**Distribution:** Europe (Austria and Italy).

**Material examined:** China, Sichuan province, Chongzhou city, 103°40′18″E, 30°40′6″N, alt. 534 m, on dead twigs of *J. regia*, 18 September 2020, F. H. Wang, WFH202009002 (SICAU 22-0090), living culture SICAUCC 22-0090.

**Note:** Our isolate is in a well-clustered clade together with four other strains of *Helminthosporium juglandinum* (Supplementary Figure 3). The nucleotide comparison of ITS, LSU, *rpb*2, and *tef*1-α (SICAUCC 22-0090) reveals high similarity to the holotype culture CBS 136922 of *H. juglandinum* [similarity = 98.3% (541/550), 0 gaps; similarity = 99.8% (852/853), 0 gaps; similarity = 98.3% (1,042/1,059), 2 gaps; similarity = 99.3% (731/736), 0 gaps, respectively]. Morphologically, the conidia of our isolate are smaller than those of the isolate CBS 136922 (89–145 × 16.5–20 μm) and have less septa. Consistent with previous reports, *H. juglandinum* is known in *Juglans* (Voglmayr and Jaklitsch, 2017), and this is the first record of this fungus from China.

#### *Helminthosporium velutinum* Link [as “*Helmisporium*”], Mag. Gesell. naturf. Freunde, Berlin 3 (1–2): 10, tab. 1:9 (1809)

**FIGURE 4:**
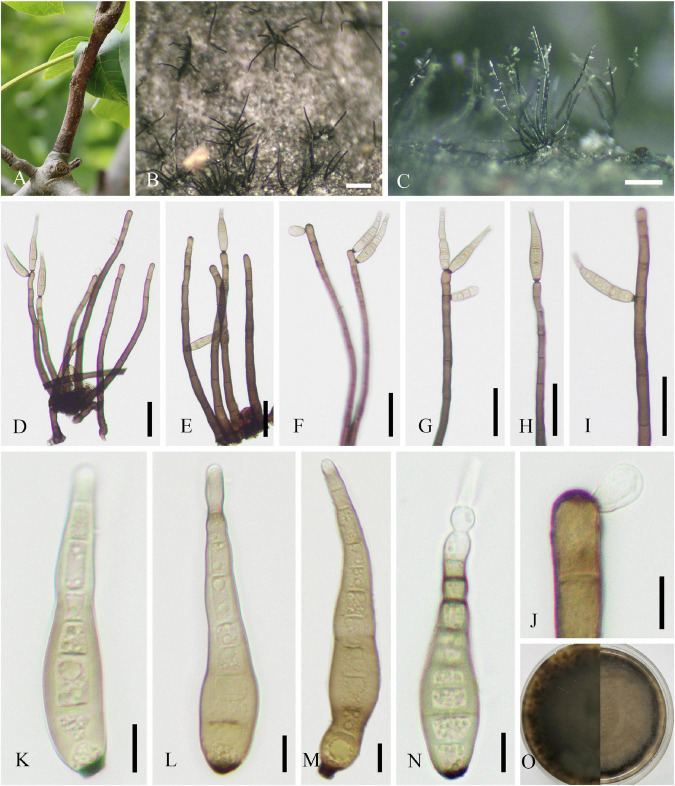
*Helminthosporium velutinum* (SICAU 22-0099). **(A)** Symptoms of dead twig observed on host. **(B)** Colonies in face view. **(C)** Conidiophores with apical and lateral conidia in side view. **(D–J)** Conidiophores with apical and lateral conidia and pores. **(K–M)** Conidia. **(N)** Germinating conidium. **(O)** Colony on PDA for 15 days. Scale bars: **(B,C)** 200 um, **(D–I)** 50 um, and **(J–N)** 10 um.

Saprobic on dead twigs of *J. regia*. **Sexual morph:** Undetermined. **Asexual morph (Figure 4):** *Mycelia* immersed and composed of branched, septate, thick-walled hyphae. *Colonies* discrete, punctiform, sometimes confluent, usually in large groups, black, and effuse. *Conidiophores* mononematous, macronematous, fasciculate, erect, unbranched, straight or flexuous, thick-walled, brown to dark brown, measuring 244–550 μm long ($\bar{x}$ = 383 μm, *n* = 20), 10–21 μm wide ($\bar{x}$ = 15 μm, *n* = 20) at the base, tapering toward apex, and 5–12 μm wide ($\bar{x}$ = 9 μm, *n* = 20) at the apex. *Conidiogenous cells* polytretic integrated, intercalary, and terminal. *Conidia* 62–91(–112) × 9–18 μm long ($\bar{x}$ = 74 × 13 μm, *n* = 20), tapering to 3–6 μm ($\bar{x}$ = 5 μm, *n* = 20) at the distal end, with a 3–7 μm ($\bar{x}$ = 5 μm, *n* = 20) wide blackish-brown scar at the base, single, obclavate, palm green to brown, apical cell paler than other cells, rounded at apex, rostrate, straight or flexuous, thick-walled, and 5–10-distoseptate.

**Culture characteristics:** Conidia germinated on PDA within 12 h, and the germ tubes were produced from both ends. Cultures grew fast on PDA, and colonies reached 6 cm in diameter after 10 days at 25°C. Colony was originally white, then brown, with thin mycelia.

**Habitat and host range:** Wide range of dead plant materials.

**Distribution:** Cosmopolitan in terrestrial ecosystems and riparian terrestrial environment.

**Material examined:** China, Sichuan province, Luding county, 102°11′34″E, 29°40′32″N, alt. 1,306 m, on dead twigs of *J. regia*, 18 July 2021, F. H. Wang, WFH202107001 (SICAU 22-0099), living culture SICAUCC 22-0100. Ibid., WFH202107008 (SICAU 22-0100), living culture SICAUCC 22-0101.

**Note:** The multigene phylogenetic analysis based on combined LSU, SSU, ITS, *rpb*2, and *tef*1-α sequence data confirm the identity of *Helminthosporium velutinum* (Supplementary Figure 3). The nucleotide comparisons of our two isolates show a high homology with the isolate MFLUCC 15–0428 from reference specimen HKAS 84015, and similarities in ITS, LSU, and SSU sequences are 99.6% (565/567; 0 gaps) and 100% (566/566; 0 gaps), 100% (804/804; 0 gaps) and 100% (804/804, 0 gaps), 100% (1,000/1,000; 0 gaps), and 100% (1,000/1,000; 0 gaps), respectively. Compared with the description of isolate MFLUCC 15–0428, our collection has shorter conidiophore (244–550 μm vs. 530–655 μm). The present study shows that our collections are the new host record of *H. velutinum* in China.

#### *Loculosulcatispora hongheensis* Wanas. J. Fungi 8: 14 (2022)

**FIGURE 5:**
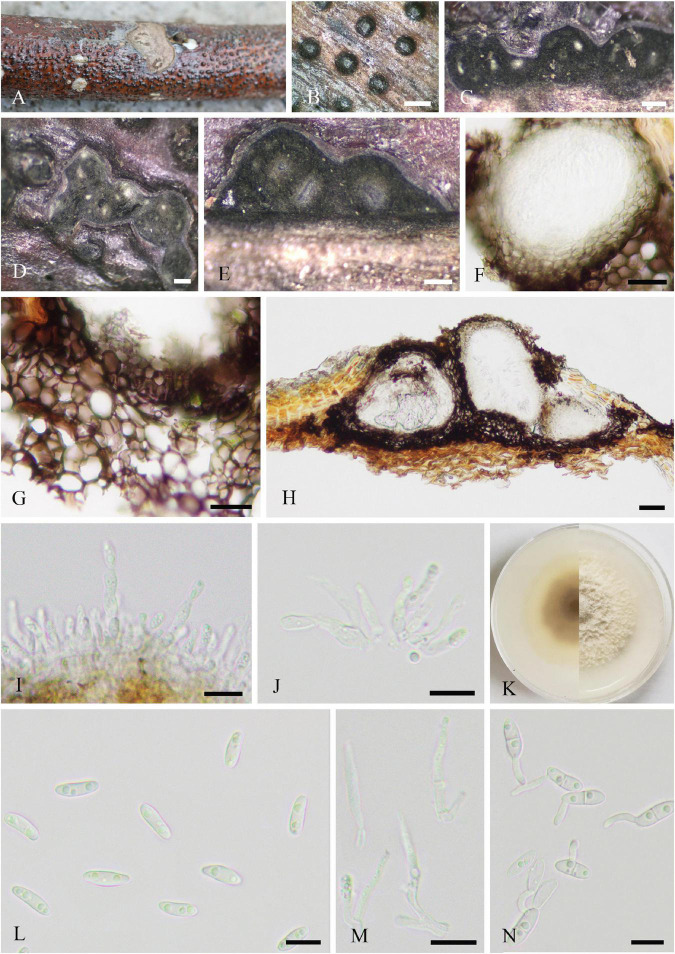
*Loculosulcatispora hongheensis* (SICAU 22-0092). **(A,B)** Conidiomata on natural wood surface. **(C–E)** Vertical and transverse sections through conidiomata. **(F,G)** Conidioma wall. **(H)** Sections through conidiomata. **(I,J)** Conidiogenous cells and developing conidia. **(K)** Culture on PDA for 15 days. **(L)** Conidia. **(M)** Paraphyses. **(N)** Germinating conidia. Scale bars: **(B)** 500 μm, **(C–E)** 200 μm, **(F–H)** 20 μm, and **(I,J,L–N)** 10 μm.

Saprobic on dead twigs of *J. regia*. **Sexual morph:** Refer to Wanasinghe et al. (2022). **Asexual morph (Figure 5):** Coelomycetous. *Conidiomata* up to 324–615 × 344–810 μm ($\bar{x}$ = 482 × 541 μm, *n* = 20), solitary, scattered, semi-immersed to superficial, commonly spherical to subglobose, rarely conical or irregular, 2–7-loculate, ostiolate, each locule 39–105 × 59–106 μm ($\bar{x}$ = 72 × 86 μm, *n* = 10), and subglobose to ellipsoidal. *Ostioles* 46–53 μm diameter, single or multiple, central, black, and papillate. *Conidiomatal walls* 15–55 μm thick, 3–7 layered, the outer layer comprising dark pigmented cells of *textura angularis*, and inner layer comprising hyaline cells of *textura angularis*. *Paraphyses* 1–3 μm wide, unconspicuous, short, sparse, mostly aseptate, occasionally 1-septate, hyaline, unbranched, and arising on inner wall of locules. *Conidiophores* reduced to conidiogenous cells. *Conidiogenous cells* enteroblastic, phialidic, discrete, doliiform to cylindrical, hyaline, aseptate, smooth-walled, and measuring 1–4 × 3–15 μm ($\bar{x}$ = 3 × 10 μm, *n* = 20). *Conidia* 3–5 × 10–15 μm ($\bar{x}$ = 4 × 12 μm, *n* = 40), 1-celled, oblong, hyaline, smooth-walled, and guttulate.

**Culture characteristics:** Conidia germinated on PDA within 12 h. Colonies reached 5 cm in diameter after 20 days at 25°C, circular, initially white and then white-gray, floccose, white and circular crack on the surface, reverse brown, darkening toward center, and white on edge.

**Habitat and host range:** Dead wood in terrestrial habitats.

**Distribution:** China.

**Material examined:** China, Sichuan province, Neijiang city, Dongxing district, 105°6′36″E, 29°48′30″N, alt. 340 m, parasitic on dead twigs of *J. regia*, 17 May 2021, F. H. Wang and C. Liu, WFH202105010 (SICAU 22-0092), living culture SICAUCC 22-0092.

**Note:** The genus *Loculosulcatispora* was introduced in *Sulcatisporaceae* not long ago based on the asexual morph characters of the typified species *L. thailandica* (Ren et al., 2020). Subsequently, *Loculosulcatispora hongheensis* was reported on dead woody litter in Yunnan, China, with the description of teleomorph (Wanasinghe et al., 2022). Our isolate is morphologically similar to the asexual description of *L. thailandica* in having multilocular pycnidia, hyaline, aseptate, oblong, and guttulate conidia. In addition, the phylogeny indicates that this isolate is in the clade of genus *Loculosulcatispora* (Supplementary Figure 4). However, our isolate has larger conidiomata, locule, conidiogenous cells, and conidia when compared with the holotype of *L. thailandica* (MFLU 20-0440) (Ren et al., 2020). Furthermore, there are obvious base-pair differences, viz. 1.79, 1.59, and 4.65% in ITS, *tef*1-α, and *rpb*2, respectively. The nucleotide comparisons of ITS, LSU, SSU, *tef*1-α, and *rpb*2 from our isolate showed a high homology with the sequences of *L. hongheensis* (HKAS122920, holotype), the similarities are 100% (692/692; 0 gaps), 100% (855/855; 0 gaps), 100% (996/996; 0 gaps), 99.6% (901/904; 1 gap), and 99.7% (933/935; 0 gaps), respectively. In this article, the anamorph of *L. hongheensis* is described for the first time.

#### *Neofusicoccum sichuanense* X. L. Xu and C. L. Yang sp. nov.

MycoBank: 845074.

Etymology: The specific epithet reflects Sichuan where the holotype was collected.

Holotype: SICAU 22-0098.

**FIGURE 6:**
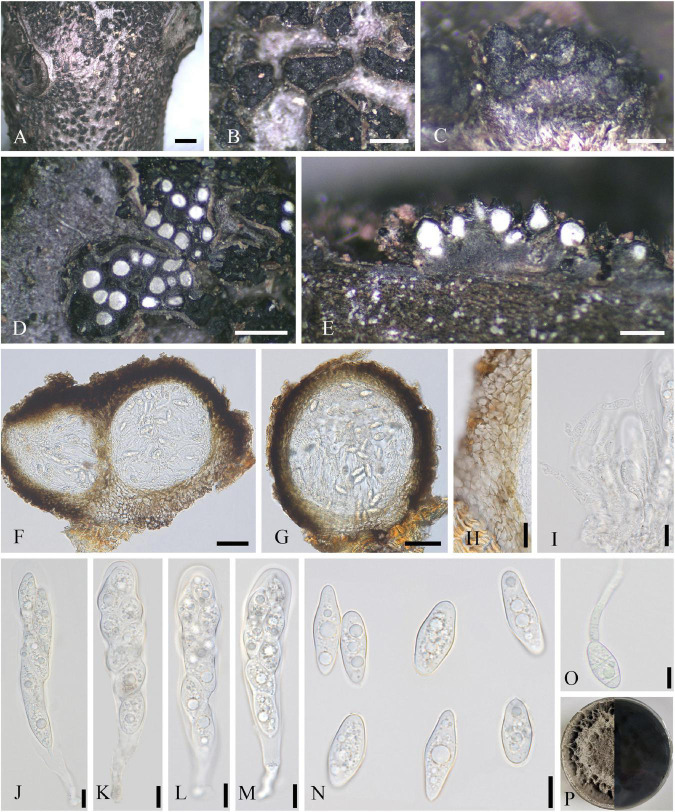
*Neofusicoccum sichuanense* (SICAU 22-0098, holotype). **(A–C)** Stromata erumpent through twig surface. **(D,E)** Vertical and transverse sections through stromata. **(F,G)** Sections through locules. **(H)** Peridium. **(I)** Pseudoparaphyses. **(J–M)** Asci. **(N)** Ascospores. **(O)** Germinating ascospore. **(P)** Culture on PDA for 15 days. Scale bars: **(A)** 2 mm, **(B,D)** 500 μm, **(C,E)** 200 μm, **(F,G)** 50 μm, **(H)** 20 μm, and **(I–O)** 10 μm.

**FIGURE 7:**
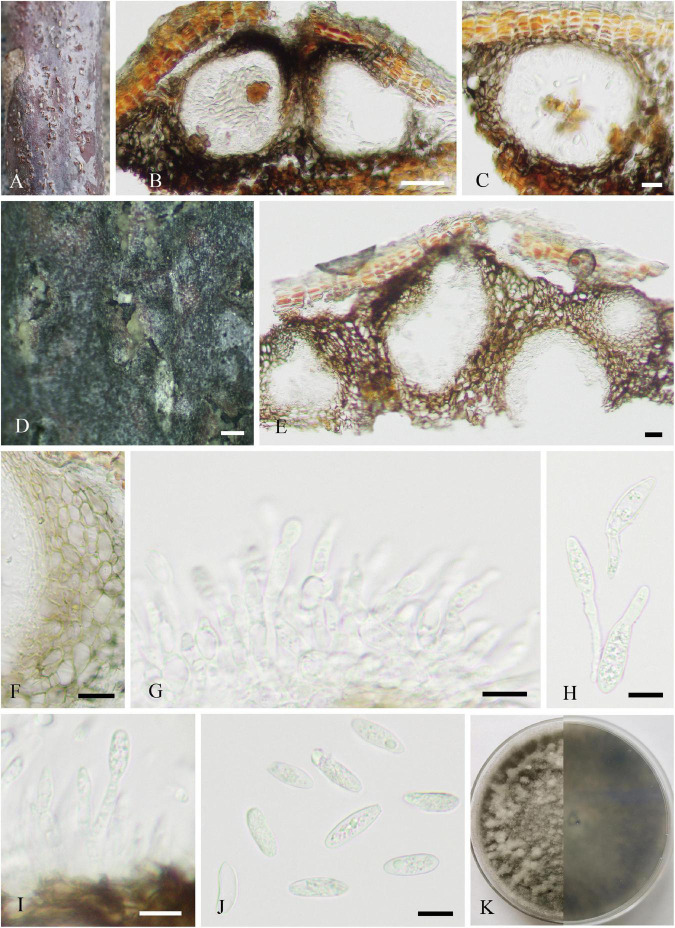
*Neofusicoccum sichuanense* (SICAU 22-0094). **(A)** Pycnidia on dead twig. **(B,C,E)** Vertical section of pycnidia. **(D)** Pycnidia with conidia mass. **(F)** Pycnidial wall. **(G,I)** Conidiophores, conidiogenous cells, and conidia. **(H)** Germinating conidia. **(J)** Conidia. **(K)** Colony on PDA for 10 days. Scale bars: **(B)** 50 μm, **(C,E,F)** 20 μm, **(D)** 200 μm, and **(G–J)** 10 μm.

Parasitic on living twigs of *J. regia*. **Sexual morph (Figure 6):** *Stromata* erumpent and well-developed, scattered or aggregated, 280–1610 μm diameter ($\bar{x}$ = 633 μm, *n* = 30), black, rounded to irregular, semi-immersed, and with multi-locule immersed in single stroma. *Ostiole* opening separately, usually papillate. *Locules* 96–220 diameter ($\bar{x}$ = 178 μm, *n* = 20), immersed in stroma, black, and circular to ovoid. *Peridium* comprising 5–11 layers of *textura angularis*, outer region of dark brown cells, and inner region of hyaline cells lining the locule. *Asci* 15–21 × 85–148 μm ($\bar{x}$ = 19 × 110 μm, *n* = 20), bitunicate, clavate, hyaline, with thin-walled, short pedicel, apically rounded, 8-spored, and forming among pseudoparaphyses. *Pseudoparaphyses* 2–6 mm broad, filiform, septate, branched, and J^–^ in Melzer’s reagent. *Ascospores* 8–13 × 17–29 μm ($\bar{x}$ = 10 × 23 μm, *n* = 40), hyaline, aseptate, thin-walled, fusoid to ellipsoid, sometimes with tapered ends, usually broadest in the middle, smooth, and granular. **Asexual morph (Figure 7):** *Conidiomata* pycnidial, epidermal, immersed, solitary or gregarious, brown to dark brown, globose to subglobose, unilocular to multilocular, with a central ostiole, becoming erumpent and exuding conidia in a white mucoid mass, and each locule 170–245 × 135–215 μm ($\bar{x}$ = 185 × 158 μm, *n* = 10). *Pycnidial walls* consisting of four to nine layers of cells, outer layer comprising dark pigmented cells of *textura angularis*, and inner layer comprising hyaline cells of *textura angularis* and *textura prismatica*. *Conidiophores* lining the inner layer of the conidioma, subcylindrical, hyaline, smooth, unbranched or branched, and 5–34 × 3–5 μm ($\bar{x}$ = 13 × 4 μm, *n* = 20). *Conidiogenous cells* hyaline, terminal, subcylindrical, rarely ampulliform, proliferating near apex, and 8–26 × 3–6 μm ($\bar{x}$ = 15 × 4 μm, *n* = 30). *Conidia* hyaline, smooth, aseptate, cylindrical to fusiform, straight or slightly curved, subtruncate to bluntly rounded at the base, acute to rounded at the apex, and 12–19 × 4–7 μm ($\bar{x}$ = 16 × 6 μm, *n* = 30).

**Culture characteristics:** Ascospores and conidia germinated on PDA within 12 h. Colonies reached 9 cm in diameter after 5 days at 25°C, circular, initially white and then gray to dark gray, floccose, and reverse dark gray to black.

**Habitat and host range:** Living twigs of *J. regia*.

**Distribution:** China.

**Material examined:** China, Sichuan province, Guangyuan city, 105°52′26″E, 36°32′28″N, alt. 557 m, on living twigs of *J. regia*, 18 May 2021, F. H. Wang, WFH202105043 (SICAU 22-0094), living culture SICAUCC 22-0094 = SICAUCC 22-0095; ibid. WFH202105041 (SICAU 22-0093), living culture SICAUCC 22-0093; ibid. WFH202105048 (SICAU 22-0095), living culture SICAUCC 22-0096; ibid. Wanyuan city, 108°7′28″E, 32°1′41″N, alt. 932 m, on living twigs of *J. regia*, 19 May 2021, F. H. Wang, WFH202105052 (SICAU 22-0096), living culture SICAUCC 22-0097; Shehong city, 105°18′54″E, 30°57′22″N, alt. 406 m, on living twigs of *J. regia*, 26 August 2021, F. H. Wang, WFH202108015 (SICAU 22-0097), living culture SICAUCC 22-0098; Chongzhou city, 103°40′18″E, 30°40′6″N, alt. 534 m, on living twigs of *J. regia*, 6 September 2021, F. H. Wang, WFH202109006 (holotype, SICAU 22-0098), ex-type living culture SICAUCC 22-0099.

**Note:** Morphologically, the asexual morphs of our collections were identical, and within our isolates, there were no nucleotide differences in ITS, *tef*1-α, *rpb*2, or *tub*2 gene regions. Therefore, we recognize that the seven isolates belong to one novel species, *N. sichuanense*, with sexual and asexual morphs and supported by phylogenetic analyses (Supplementary Figure 5). Phylogenetically, our strains are monophyletic in a clade, having a sister relationship to *N. hyperici* with weak bootstrap (56% ML/0.95 PP). In the comparison of ITS, *tef*1-α, *rpb*2, and *tub*2 sequences, the type of strain of the novel species (SICAUCC 22-0099) shows relatively high similarities to that of *N. hyperici* (MUCC 241, holotype), viz. 98.5% (471/478; 7 gaps), 96.7% (211/218; 6 gaps), 100% (588/588; 0 gaps), and 100% (370/370; 0 gaps), respectively. However, the encoding genes of *rpb*2 and *tub*2 in *N. hyperici* (MUCC 241) are shorter than that in our isolates. Nevertheless, this new species differs from *N. hyperici* in having multilocular conidiomata, obvious conidiophores, and larger conidiogenous cells (8–26 × 3–6 μm vs. 4.4–7 × 1.6–2.3 μm), while *N. hyperici* has unilocular conidiomata and simplified conidiophores.

#### *Ophiognomonia leptostyla* (Fr.) Sogonov, Stud. Mycol. 62: 62 (2008)

**FIGURE 8:**
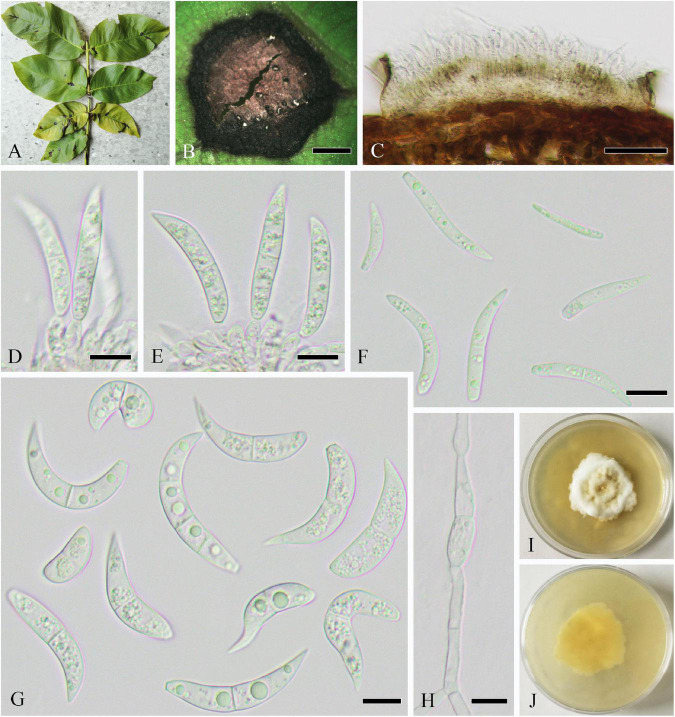
*Ophiognomonia leptostyla* (SICAU 22-0102). **(A,B)** Symptoms of leaf spots observed on host. **(C–E)** Conidiophores, conidiogenous cells, and developing conidia. **(F)** Microconidia. **(G)** Macroconidia. **(H)** Germinating conidium. **(I,J)** Colonies on PDA for 25 days. Scale bars: **(B)** 1,000 μm, **(C)** 50 μm, and **(D–H)** 10 μm.

Parasitic on leaf of *J. regia*, *J. sigillata*, and *J. regia* × *J. sigillata*, and causing brown leaf spots. **Sexual morph:** Undetermined. **Asexual morph (Figure 8):** Coelomycetous. *Acervuli* 85–350 × 80–210 μm ($\bar{x}$ = 190 × 136 μm, *n* = 30), growing under the leaf epidermis, rupturing when mature, dark brown to black, usually with an opening, discoid, and single-chambered. *Conidiophores* reduced to conidiogenous cells. *Conidiogenous cells* 7–15 × 2–6 μm ($\bar{x}$ = 12 × 4 μm, *n* = 30), hyaline, cylindrical or ampulliform, monophialidic, rarely branch-like phialidic, and enteroblastic. *Macroconidia* 17–48 × 4–8 μm ($\bar{x}$ = 34 × 6 μm, *n* = 50), lunate, reniform, basal cell rounded, apical cell with acute end, uniseptate, occasionally 2–3-septate, sometimes constricted at septum, and hilum usually conspicuous. *Microconidia* 14–30 × 2–5 μm ($\bar{x}$ = 22 × 3 μm, *n* = 30), cylindrical, botuliform or lunate, ends rounded, occasionally acute at apical cell, 0–1-septate, and hilum sometimes conspicuous.

**Culture characteristics:** Colonies on PDA attaining 30–50 mm diameter in 4 weeks, dense, subcircular, crenated, initially white, gradually turning yellowish with black spots with abundant sporulation, aerial mycelia developed and fluffy, reverse pale yellow or brown, and pale yellow at the margin.

**Habitat and host range:** Leaves of *Juglans* spp., *Pterocarya* spp., and *Carya* spp. (Juglandaceae) causing anthracnose and leaf blotch.

**Distribution:** Austria, Bulgaria, Canada, China, Germany, Iran, Italy, Korea, Portugal, Poland, Russia, Spain, Switzerland, South Africa, and United States.

**Material examined:** China, Sichuan province, Dazhou city, Wanyuan city, 105°18′34.20″E 30°57′52.33″N, alt. 362 m, on leaves of *J. regia*, 22 April 2020, H. B. Yang and C. L. Yang, YHB202004003 (SICAU 22-0102), living culture SICAUCC 22-0103; ibid. Mianyang city, 105°25′55.1″E, 31°5′4.6″N, alt. 368 m, from leaves of *J. regia*, 22 April 2020, H. B. Yang and C. L. Yang, YHB202004004 (SICAU 22-0103), living culture SICAUCC 22-0104.

**Note:** Based on the morphological observations, our collections showed identical characteristics, and were similar to the asexual descriptions of *O. leptostyla* provided by Walker et al. (2012b). The morphological comparison showed that the macroconidia and microconidia of our isolates were generally larger than those described by Walker et al. (2012b). *O. leptostyla* is the prevalent causal agent of walnut anthracnose and leaf blotch in the United States, South America, Europe, and Asia (Neely and Black, 1976; Juhásová et al., 2006; Belisario et al., 2008). The strains clustered with the strains of *O. leptostyla* with high bootstrap support (100% ML/1 PP) (Supplementary Figure 6). *O. leptostyla* has been prviously reported as pathogen of leaf spot on *J. sigillata* and *J. regia* × *J. sigillata*, it might be a common fungus on Juglans causing leaf spot in Sichuan province.

#### *Periconia byssoides* Pers., Syn. Meth. Fung. 2: 686 (1801)

**FIGURE 9:**
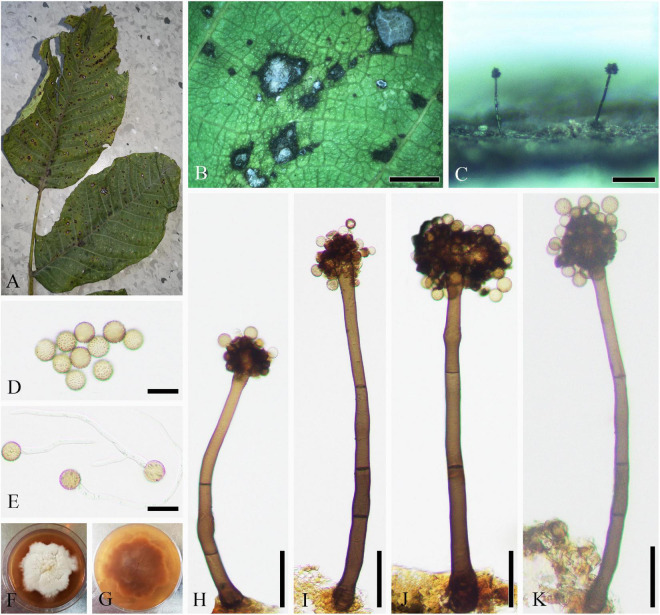
*Periconia byssoides* (SICAU 22-0106). **(A,B)** Symptoms of leaf spots observed on host. **(C)** Close-up of colonies on substrate. **(D)** Conidia. **(E)** Germinating conidia. **(F,G)** Colonies on PDA for 25 days. **(H–K)** Conidiophores. Scale bars: **(B)** 2,000 μm, **(C)** 200 μm, **(D,E)** 20 μm, and **(H–K)** 50 μm.

Parasitic on living leaves causing leaf spots or associated with twig dieback of *J. regia*. On host, spots circular or irregular, initially brown to dark brown, expanding and becoming white speckle in the late, and dark brown at the margin. **Sexual morph:** Undetermined. **Asexual morph (Figure 9):** Hyphomycetous. *Mycelia* not observed. *Conidiophores* 240–330 × 13–19 μm ($\bar{x}$ = 297 × 16 μm, *n* = 15), macronematous, mononematous, single, cylindrical, brown to dark brown, erect, or curved, 1–3-septate, thick-walled, and smooth. *Conidiogenous cells* monoblastic, cylindrical, sometimes swollen region partly, and discrete on stipe. *Conidia* 11–15 μm ($\bar{x}$ = 13 μm, *n* = 50) diameter, catenate, globose, aseptate, initially hyaline to pale brown, brown to dark brown upon maturity, thin-walled, and verruculose.

**Culture characteristics:** Conidia germinated on PDA within 12 h. Colonies on PDA reach 6 cm in diameter after 30 days, medium dense, initially circular and white, later undulate, with irregular margin, floccose, generating red pigment inside the medium, and initially reverse rosy but later black.

**Habitat and host range:** Saprobic or associated with leaf and stem spots, blight, twig dieback, and fruit mold on various hosts from multiple genera in multiple families.

**Distribution:** Cosmopolitan.

**Material examined:** China, Sichuan province, Guangyuan city, 106°10′43.81″E, 32°12′30.59″N, alt. 485 m, on leaves of *J. regia*, 11 July 2020, H. B. Yang and C. L. Yang, YHB202007001 (SICAU 22-0106), living culture SICAUCC 22-0107; Neijiang city, 105°6′36″E,29°48′30″N, alt. 340 m, on leaves of *J. regia*, 17 May 2021, F. H. Wang and C. Liu, WFH202105011 (SICAU 22-0104), living culture SICAUCC 22-0105; Wanyuan city, 108°7′28″E, 32°1′41″N, alt. 932 m, on decaying twigs of *J. regia*, 19 May 2021, F. H. Wang, WFH202105053 (SICAU 22-0105), living culture SICAUCC 22-0106.

**Note:** Our collections were morphologically similar to *P. byssoides* in having macronematous, mononematous, unbranched, erect, and light brown to dark brown conidiophores, monoblastic, ovoid to globose conidiogenous cells, and globose to subglobose, light brown to dark brown, verruculose, aseptate conidia (Jayasiri et al., 2019), which is also supported by phylogenetic trees (Supplementary Figure 7). Consistent with the conclusion made by Yang et al. (2022), the morphological comparison of *P. byssoides* strains isolated from different hosts showed a slight variation in the size of conidiophores and conidia. Compared with previous reports, the conidiophores of our collections are especially shorter and wider (Markovskaja and Kačergius, 2014; Jayasiri et al., 2019; Tennakoon et al., 2021; Yang et al., 2022). In this article, three isolates of *P. byssoides* are obtained from leaves and twigs of *J. regia*, and it is reported as a pathogen on *J. regia* in Sichuan, China for the first time.

#### *Rhytidhysteron subrufulum* X. L. Xu and C. L. Yang, Cryptogamie, Mycologie 43: 72 (2022)

**FIGURE 10:**
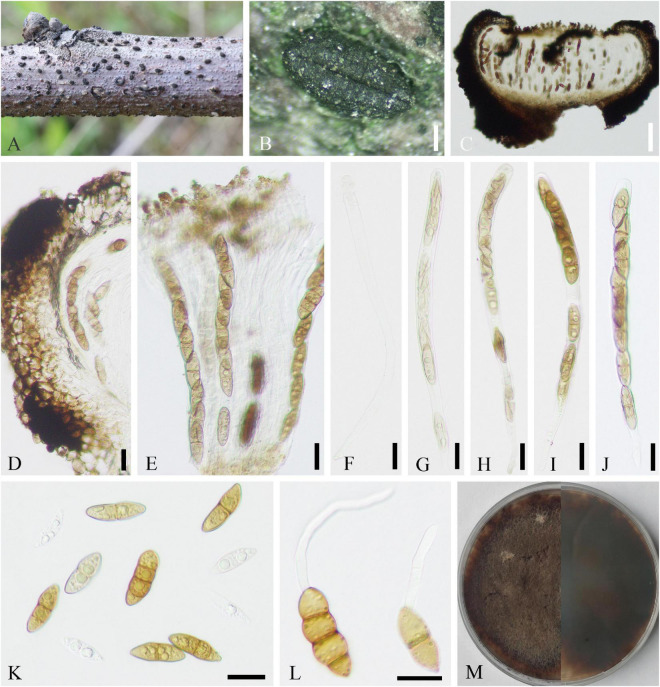
*Rhytidhysteron subrufulum* (SICAU 22-0091). **(A,B)** Ascomata on host substrate. **(C)** Vertical section of ascoma. **(D)** Exciple. **(E)** Pseudoparaphyses and asci. **(F–J)** Asci. **(K)** Ascospores. **(L)** Germinating ascospores. **(M)** Colony on PDA for 90 days. Scale bars: **(B)** 200 μm, **(C)** 100 μm, and **(D–L)** 20 μm.

Saprobic on dead twigs of *J. regia*. **Sexual morph (Figure 10):** *Ascomata* 400–1,400 μm long × 440–600 wide × 460–700 high ($\bar{x}$ = 1,050 × 550 × 540 μm, *n* = 10), apothecioid, carbonaceous, scattered to gregarious, black, labiates and elliptic or irregular in shape, perpendicularly striate along the long axis, and reddish brown to black on the disk. *Exciple* 26–69 μm wide ($\bar{x}$ = 54, *n* = 10), two-layered, outer layer comprising thick-walled, brown to hyaline cells of *textura angularis* and *textura globulosa*, and inner layer comprising thin-walled, light brown to hyaline cells of *textura angularis* and *textura prismatica*. *Hamathecium* composed of 1–3 μm wide at the base and 2–4 μm wide at swollen tips (*n* = 20), dense, and septate pseudoparaphyses, branched, and forming brown epithecium above the asci, slightly swollen at the apex, and hymenium turns blue in Melzer’s reagent, J^+^. *Asci* 170–280 × 11–14 μm ($\bar{x}$ = 240 × 13 μm, *n* = 15), 8-spored, bitunicate, clavate to cylindrical, with short pedicel and apically rounded with an ocular chamber, and J^–^ in Melzer’s reagent. *Ascospores* 24–36 × 9–14 μm ($\bar{x}$ = 32 × 13 μm, *n* = 30), ellipsoidal or fusiform, straight or slightly curved, slightly pointed at both ends, partially overlapping, uniseriate, 1–3-septate, constricted septum, light brown to dark brown, and without a mucilaginous sheath. **Asexual morph:** Undetermined.

**Culture characteristics:** Ascospores germinated on PDA within 24 h, and germ tubes were produced from any cell. Colonies grew on PDA and reached 3 cm in diameter after 8 days at 25°C, flat, circular, initially white, and gradually becoming yellow to gray, dark gray.

**Habitat and host range:** Dead woody plants.

**Distribution:** China.

**Material examined:** China, Sichuan province, Chongzhou city, 103°40′18″E, 30°40′6″N, alt. 534 m, from dead twigs of *J. regia*, 14 January 2021, F. H. Wang, WFH202101003 (SICAU 22-0091), living culture SICAUCC 22-0091.

**Note:** *Rhytidhysteron* species had a wide distribution and host range in Sichuan province, and were mainly identified based on multigene phylogeny (Xu et al., 2022). The isolate was identified as *Rhytidhysteron subrufulum* by the similar morphological characteristics and multigene phylogenetic analysis based on combined LSU, SSU, ITS, and *tef*1-α sequence data (Supplementary Figure 8). Compared with the holotype SICAU 19-0010, this isolate has smaller ascomata ($\bar{x}$ = 1,909 × 1,220 × 546 μm) but longer asci ($\bar{x}$ = 202 × 16 μm). In addition, this specimen is similar to the previous collection SICAU 19-0009 in a large number of fusiform and 1-septate ascospores that obviously pointed at both ends. However, the difference is that the ascospores from this collection could germinate at room temperature within 12 h, while the other did not for a week. In the represent study, *J. regia* was confirmed as a new host record for *R. subrufulum*.

#### *Sphaerulina juglandina* X. L. Xu and C. L. Yang sp. nov.

MycoBank: 845075.

Etymology: The specific epithet reflects *Juglans*, from which host the holotype was collected.

Holotype: SICAU 22-0108.

**FIGURE 11:**
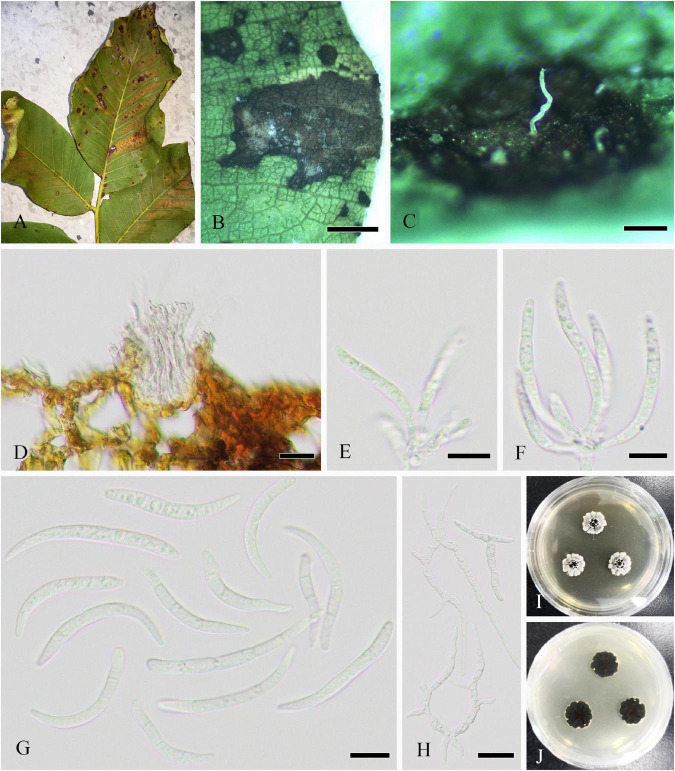
*Sphaerulina juglandina* (SICAU 22-0108, holotype). **(A,B)** Symptoms of leaf spots observed on host. **(C)** Close-up of conidiomata. **(D)** Section though conidioma. **(E,F)** Conidiogenous cells and conidia. **(G)** Conidia. **(H)** Germinating conidia. **(I,J)** Colonies on PDA for 15 days. Scale bars: **(B)** 2,000 μm, **(C)** 200 μm, **(D)** 20 μm, **(E–G)** 10 μm, and **(H)** 20 μm.

Parasitic on leaves of *J. regia*, causing leaf spots on host, spots irregular, and initially brown to dark brown but expanding and becoming dark brown speckle later. **Sexual morph:** Undetermined. **Asexual morph (Figure 11):** Coelomycetous. *Conidiomata* 45–95 × 38–71 μm ($\bar{x}$ = 72 × 50 μm, *n* = 15), flat stromatic, amphigenous, solitary, scattered, initially immersed, finally erumpent, usually broke through the upper epidermis and extravasated conidia, tiny dots though epiphyllous, lacte. *Stromatal base* 3–10 μm thick, and composed of several layers of cells of hyaline. *Conidiophores* reduced to conidiogenous cells or giving rise to several terminal conidiogenous cells, hyaline, cylindrical, smooth-walled, and aseptate. *Conidiogenous cells* 3–9 × 2–4 μm ($\bar{x}$ = 7 × 3 μm, *n* = 20), hyaline, subcylindrical, sometimes slightly tapered toward the apex, monophialidic, and with apical loci indistinctly. *Conidia* 26–49 × 2–4 μm ($\bar{x}$ = 37 × 3 μm, *n* = 50), cylindrical, mostly curved or flexuous, narrowly or broadly rounded at the apex, narrowed slightly to the truncate base, 1–4(–5)-septate, not or slightly constricted around the septum, hyaline, and contents with few oil droplets and minute granules in each cell.

**Culture characteristics:** Colonies on PDA attaining 2 cm diameter in 30 days, slow-growing, subcircular, raised, radially striated with lobate edge, black droplets formed on the surface with large droplets in the center, grayish white, and reverse dark brown to black.

**Habitat and host range:** Living leaves of *J. regia*.

**Distribution:** China.

**Material examined:** China, Sichuan province, Guangyuan city, 106°10′43.81″E, 32°12′30.59″N, alt. 485 m, from leaves of *J. regia*, 11 July 2020, H. B. Yang and C. L. Yang, YHB202007002 (holotype, SICAU 22-0108), ex-type living culture SICAUCC 22-0109; ibid. Shehong city, 105°18′54″E, 30°57′22″N, alt. 406 m, from leaves of *J. regia*, 17 May 2021, F. H. Wang, WFH202105021 (SICAU 22-0107), living culture SICAUCC 22-0108.

**Note:** Our isolates were shown to have *Septoria*-like asexual morphs as described by Quaedvlieg et al. (2013). In the same year, Quaedvlieg et al. (2013) and Verkley et al. (2013) provided descriptions of most *Sphaerulina* species based on the sexual morph and *Septoria*-like asexual morphs, which were either endophytes or important plant pathogens. Compared with previous reports, this species can be distinguished by having less conidial septa, smaller conidia than generally seen in most species, or having difference in appendage, such as *Sp. pseudovirgaureae* [3–10-septate, 40–60(–80) × 2.5 μm], *Sp. oxyacanthae* (6–12-septate, with appendage), *Sp. gei* [(0–)2–5(–8)-septate, 33–65 × 2–2.8 μm]. Furthermore, although it has conidia that resemble those of *Sp. cornicola* [1–3(–5)-septate, 24–40 × 3–4 μm] and *Sp. hypericin* [1–3(–5)-septate, 24–55 × 2.5–3.5 μm], our two isolates are phylogenetically distinct in a monophyletic clade (Supplementary Figure 9). Based on the phylogeny, the novel species and another species, *Sp. abeliceae*, are distinct from other species in two distinctly independent lineages within the genus *Sphaerulina*, in which our two isolates are clustered together with strong support (100% ML/1 PP). Hence, we established a new species, *Sp. juglandina*, to accommodate our isolates. Presently, there are no species of *Septoria*-like fungi known from *Juglans*.

### Pathogenicity test

**FIGURE 12:**
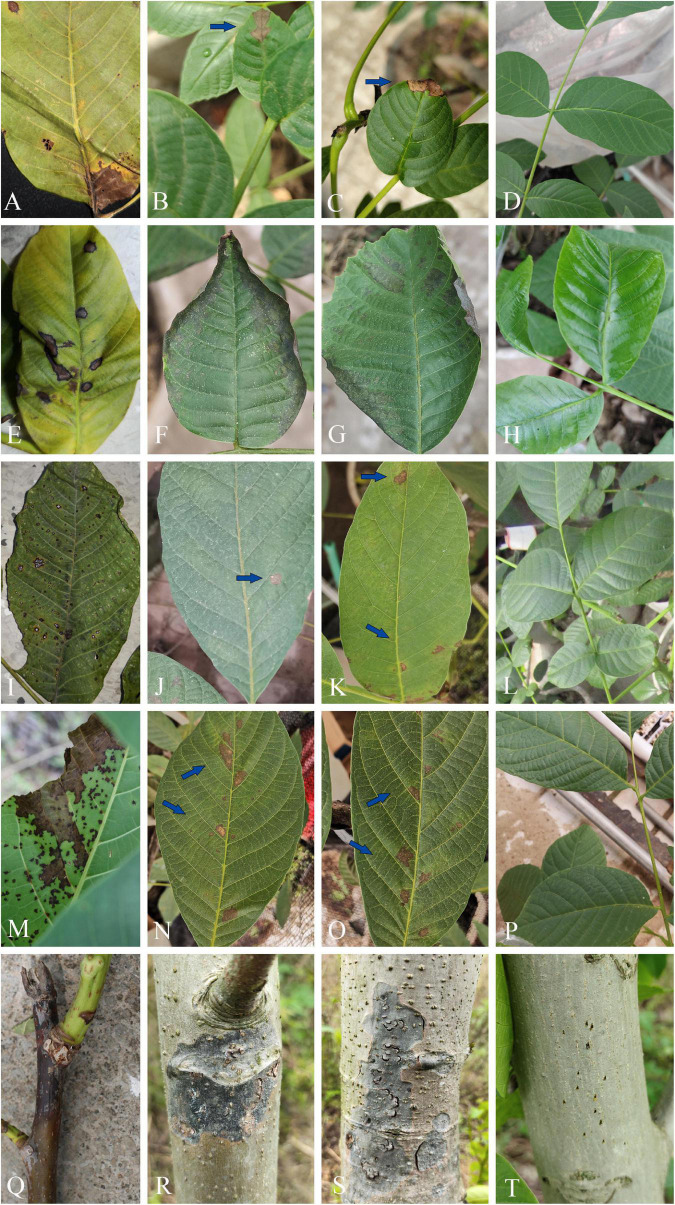
Leaf spot and stem blight symptoms on *Juglans regia* caused by pathogenic fungi. **(A–D)** Leaf necrosis symptoms caused by *Cladosporium tenuissimum* (SICAUCC 22-0110): **(A)** Typical symptoms in natural state and **(B)** necrotic lesions (blue arrow) after 20 days of inoculation. **(E–H)** Leaf necrosis symptoms caused by *Ophiognomonia leptostyla* (SICAUCC 22-0103): **(E)** Typical symptoms in natural state and **(F,G)** necrotic lesions after 15 days of inoculation. **(I–L)** Leaf necrosis symptoms caused by *Periconia byssoides* (SICAUCC 22-0107): **(I)** Typical symptoms in natural state and **(J,K)** necrotic lesions (blue arrow) after 25 days of inoculation. **(M–P)** Leaf necrosis symptoms caused by *Sphaerulina juglandina* (SICAUCC 22-0108): **(M)** Typical symptoms in natural state and **(N,O)** necrotic lesions (blue arrow) after 25 days of inoculation. **(Q–T)** Blight symptoms caused by *Neofusicoccum sichuanense* (SICAUCC 22-0094): **(Q)** Typical symptoms in twigs in natural state and **(R,S)** stem blight symptoms after 40 days of inoculation. **(D,H,L,P,T)** Blank control group, no symptoms on leaves and stems after 25 days of inoculation.

By pathogenicity test, the infection occurred at inoculation sites on leaves or branches (Figure 12). All the four species of leaf pathogens, viz. *O. leptostyla*, *P. byssoides*, *Sp. juglandinum*, and *Cl. tenuissimum*, caused chlorotic spots on the inoculated sites after 5 days of inoculation, and then the spots gradually expanded and turned into brown necrotic lesions on the leaves after 10–15 days of inoculation. The last species, *N. sichuanense*, caused pale brown lesions at the inoculated sites after 3 days of incubation, and then the individual lesion area enlarged and coalesced into obvious black necrotic patches after 30 days. No symptoms were observed in the non-inoculated controls. The morphology and DNA sequences of isolates reisolated from the infected tissue by tissue isolation were consistent with those of isolates for inoculations. Koch’s postulates were completed by the successful reisolation of fungal isolates from the infected tissue inoculated with the five species.

## Discussion

In the present study, we described 10 species in Dothideomycetes and Sordariomycetes associated walnut trees. Within the broader region of China, there are reports of partial fungi on other known or undescribed plants (Zhang, 2003; Zhu et al., 2016; Jayawardena et al., 2018; Long et al., 2021; Farr and Rossman, 2022; Wanasinghe et al., 2022; Xu et al., 2022; Yang et al., 2022). To our knowledge, these are the first accounts of these species in Sichuan province. Furthermore, *C. tenuissimum*, *D. vulgaris*, *L. hongheensis*, *N. sichuanense*, *P. byssoides*, *R. subrufulum*, and *S. juglandina* are first recorded on *J. regia*.

The walnut disease caused by fungi is an important factor in the production and management of walnut and has been restricting the development of the walnut industry. The related studies were mainly reported in Asia, Europe, and North America (Chen et al., 2014; Scotton et al., 2015; Wang et al., 2020). Roughly estimated, there were more than 100 related bodies of literature, mostly relating to diseases occurrence and control, pathogen identification, biological characteristics, and fungicide screening for control, and a few on disease resistance evaluation and walnut breeding in China. And the studies of walnut related fungi have focused on pathogens, endophytes, and rhizosphere fungi (Ju et al., 2015; Mao et al., 2016; Yang et al., 2021). At present, there are about 22 fungal diseases of walnut trees, and more than 130 species of pathogenic fungi have been recorded worldwide. The pathogens mainly belong to Ascomycota and Basidiomycota, among which the ascomycetes were more than 110 species (Dai et al., 2007; Chen et al., 2014; Fan et al., 2015a,b, 2018; Eichmeier et al., 2020; Jayawardena et al., 2020). However, most of the studies were focused on a particular disease caused by different fungi. For example, walnut twig blight was caused by various fungi, viz. *Neofusicoccum parvum*, *Phomopsis juglandina*, *Colletotrichum godetiae*, and unidentified species in Diaporthaceae and Botryosphaeriaceae (Thomidis et al., 2011; Cheon et al., 2013; Sun, 2013; López-Moral et al., 2020; Varjas et al., 2021). In addition, *J. regia* was usually infected by a range of canker disease pathogens, which often cause serious losses (Fan et al., 2015b). Similarly, walnut anthracnose pathogens were also diverse (Wang et al., 2017, 2018, 2020; Varjas et al., 2021). There are 17 most common fungal diseases occurring on leaves, twigs, and fruits of walnut trees in China, including more than 70 species of fungi, which are mostly ascomycetes (Zhang et al., 2010; Li et al., 2015, 2017; Wang et al., 2017; Fan et al., 2018, 2020; Liu et al., 2020; Yang et al., 2021a,b). Most fungi isolated from walnut trees were recorded in the general literature, lacking living culture and molecular data. Furthermore, the research on fungal diseases of walnut are inconsistent in different areas, and more studies are reported in partial regions of Guangxi, Yunnan, Shandong, Henan, Hebei, and Shanxi provinces. There is a lack of research on fungal diseases in Sichuan province where the introduced cultivars are exposed to high-temperature, high-humidity, and low-sunshine stresses, resulting in weak trees, severe fruit drop, or fruitlessness. By literature review, approximately 20 species of pathogenic fungi have been recorded; among them, about 10 species were identified with certain evidence, but sufficient morphological characters and molecular data are still lacking (Table 1). Therefore, it is not enough to support the identification of the fungi, and the existing species need to be recollected, epitypified, and sequenced. Because of the wide distribution of walnut trees and weak research on Sichuan province, the diversity of ascomycetes is still underestimated.

**TABLE 1:** Pathogenic fungi associated with walnut trees (*Juglans*, Juglandaceae) with certain evidence in Sichuan province.

Pathogen	Disease	Morphological characteristics	Molecular data (absent)	Host	City	Literature
*Alternaria alternata*	Walnut brown spot	On potato dextrose agar (PDA): conidia av. 25 × 7.5 μm, inverted pear-shaped, with a short beak, 1–4 diaphragms, 0–3 mediastinum	ITS, gapdh (*tef*1-α, *ATPase*, *rpb*2)	*J. regia*	Deyang City	Yang et al., 2017
*Diaporthe eres*	Branch blight	Undefined	ITS, *tef*1-α, *tub*2, *his*3, *cal* (–)	*J. regia*	Guangyuan City	Fan et al., 2018
*Fusarium fujikuroi*	Stem rot	On synthetic low nutrient agar (SNA): microconidia 2.2–3.8 × 7.6–11.7 μm, oval, elliptic or clavate, zero septate; conidiophores branched or unbranched, solitary or in groups, phialides cylindrical to flask-shaped, monophialidic and polyphialidic; macroconidia 2.1–3.9 × 26.2–53.4 μm, long and slender with a curved apical cell and foot-like basal cell, 3–4-septate	ITS, LSU, *tef*1-α, *tub*2 (*rpb*1, *rpb*2, *cmdA*)	*J. sigillata*	Zigong City	Han et al., 2021
*Fusarium solani*	Root rot	On carnation leaf-piece agar (CLA): microconidia av. 10.6 × 9.1 μm, ovoid, no septa or one septum; macroconidia av. 47.4 × 5.3 μm, 1–3-septate, sickle-shaped; chlamydospores av. 10.3 × 9.2 μm, single or paired, circular to ovate, smooth or not smooth	ITS, *tef*1-α (*rpb*2)	*J. sigillata*	Liangshan Prefecture	Zheng et al., 2015
*Juglanconis appendiculata*	Branch blight	On the host: conidiomata 0.3–0.7 mm, acervular, black and scattered; conidiophores 30–43 × 3–8 μm, narrowly cylindrical, simple or branched at the base; conidiogenous cells annellidic with distinct annellations; conidia 17–32 × 7–12 μm, unicellular, brown, narrowly ellipsoid with gelatinous sheaths and truncate scars at the base	ITS, *ms*204, *tef*1-α, *tub*2 (–)	*J. sigillata*	Mianyang City	Wang et al., 2022b
*Lasiodiplodia pseudotheobromae*	Trunk canker	On the host: conidiomata 160–280 × 140–190 μm, stromatic, uniloculate, dark brown to black, immersed, and erumpent; pycnidial walls 32–58 μm wide, 5–7 layered with brown to dark brown cells; conidia 21.5–31 × 11.5–15.7 μm, hyaline, ellipsoidal with rounded apex and base, widest at the middle, thick-walled, and unicellular	ITS, LSU, SSU, *tef*1-α, *tub*2 (–)	*J. sigillata*	Chongzhou City	Wang et al., 2022c
*Neofusicoccum parvum*	Branch rot	On PDA: conidia 15.2–17.2 × 4.6–6.4 μm, circular, elliptic, or irregular, aseptate	ITS (LSU, *tef*1-α, *tub*2, *rpb*2)	*J. regia*	Ya’an City, Mianyang City	Yin and Zhu, 2016
*Ophiognomonia leptostyla*	Brown leaf spot	On PDA: conidiogenous cells subcylindrical to cylindrical, or ampulliform, hyaline, rarely branched; macroconidia 22–40.5 × 2.5–8.3 μm, lunate, reniform, hyaline, 1–3-septate, constricted at the septum, the basal cell rounded, the apical cell with an acute end; microconidia 10–28.5 × 1.9–3.7 μm, botuliform or subfusiform, hyaline, both ends rounded, straight or curved, aseptate	ITS, *ms*204, *tef*1-α (–)	*J. sigillata*, *J. regia* × *J. sigillata*	Guangyuan City, Dazhou City	Yang et al., 2021a,b
*Palmiascoma qujingense*	Branch blight	On the host: ascostroma 260–410 × 210–320 μm, black, globose to subglobose, short-papillate, ostiolate; asci 55–78 × 8–12 μm, 8-spored, bitunicate, cylindrical, short pedicellate; ascospores 12–17 × 3–5 μm, 1-septate, fusiform to ellipsoidal, slightly curved, guttulate On PDA: conidiomata 220–300 × 240–380 μm, black, globose to subglobose; conidia 3–7 × 2–4 μm, oblong to ellipsoidal, aseptate and smooth-walled	ITS, LSU, SSU, *rpb*2	*J. regia*	Chongzhou City	Wang et al., 2022a
*Phellinus igniarius*	White rot of heartwood	Absent	- (ITS)	*Juglans* spp.	Undefined	Dai et al., 2007
*Phomopsis capsici*	Twig blight	On PDA: α-conidia 5.6–9.2 × 1.0–2.2 μm, aseptate, ovoid to oval; β-conidia 21.6–30.4 × 0.2–0.4 μm, aseptate, filiform, curved	ITS (*tef*1-α, *his*3, *cal*, *tub*2)	*J. regia*	Guangyuan City	Li et al., 2017
*Phyllactinia juglandis*	Powdery mildew	On the host: perithecia 166–239 μm; appendices 104–339 μm, straight or slight curving; asci 59–103 × 24–45 μm, long elliptic or ovate, 2-spored; ascospores 29–44 × 17–25 μm, elliptic, rectangular, or ovate	- (ITS)	*J. regia*	Undefined	Yu and Lai, 1979

On the basis of our investigation, partial pathogenic fungi on walnuts have been recorded in Sichuan. *Palmiascoma qujingense*, *Lasiodiplodia pseudotheobromae* (Dothideomycetes), and *Juglanconis appendiculata* (Sordariomycetes) causing branch blight in *J. regia* and branch canker and branch blight in *J. sigillata* have been documented in this region (Wang et al., 2022a,b,c). In addtion, a species of *Ragnhidiana* (Dothideomycetes) causing leaf spots on *J. regia* have also been verified by pathogenicity test (articles in press). The results of this study, combined with previous reports, showed that *O. leptostyla* was a common pathogen in various species of walnut trees (Yang et al., 2021a,b), viz. “Chuanzao 2,” a variety derived from hybridization of *J. regia* and *J. sigillata* and distributed in rural regions of southwest China, “Mianhe 1” and “Wumizi,” the cultivated species of *J. regia*, and highly resistant clones of *J. sigillata*. Thus, this finding highlights the occurrence of *O. leptostyla* that cause losses in walnut cultivation. *C. tenuissimum* is a quite common saprobic species isolated from numerous substrates, and is the agent of leaf spot or blight disease in China (Han et al., 2019; Li et al., 2021; Xie et al., 2022; Zhou et al., 2022). However, the isolates of *P. byssoides*, which have been verified based on molecular data, are saprobic on host plants in Vitaceae, Apiaceae, Cornaceae, Cannabaceae, Euphorbiaceae, Magnoliaceae, Fabaceae, and Rosaceae (Yang et al., 2022). Other isolates associated with leaf and stem spots, blight, twig dieback, and fruit mold were documented in the earlier literature (Farr and Rossman, 2022). *Neofusicoccum* is a genus of endophytes and pathogens in the family Botryosphaeriaceae, which is known for causing dieback symptoms and cankers in woody hosts (Tennakoon et al., 2021). Members of *Neofusicoccum* have a cosmopolitan geographic distribution and a wide host range, including numerous wild and ornamental species, as well as economically important hosts in agriculture, horticulture, and forestry. Similarly, *Sphaerulina* species usually cause leaf spots on various plant substrates in the families Asteraceae, Betulaceae, Caprifoliaceae, Ericaceae, Fagaceae, Pinaceae, Rosaceae, Sapindaceae, Salicaceae, and Ulmaceae (Quaedvlieg et al., 2013; Crous et al., 2020). In the present study, to conduct Koch’s postulates, healthy *J. regia* plants were inoculated with a spore suspension or a mycelium plug onto the corresponding tissues. To our knowledge, the known species *C. tenuissimum* and *P. byssoides*, and novel the species *N. sichuanense* and *Sphaerulina juglandina* were first known as pathogens infecting a particular tissue of *J. regia*, causing leaf spot and twig dieback. In view of the diversity of pathogen, appropriate measures should be taken to control walnut diseases in walnut-planting areas in Sichuan.

Fungi are abundant in plant culms and leaves, and the number of saprophytic fungi is more than that of pathogenic fungi, as shown in studies on bambusicolous fungi (Dai et al., 2016; Zeng et al., 2022). During our investigation, more than 300 specimens have been collected, from which about 200 isolates were obtained. The preliminary classification of identifiable species showed that the isolates belong to classes Dothideomycetes (55%) and Sordariomycetes (41%), and that saprophytes (35%) were less abundant than pathogens (64%). Besides, the isolates from twig (62%) are more than those from leaf (18%), branch (13%), fruit (4%), and other tissues. In this study, we described five fungi, *D. vulgaris*, *R. subrufulum*, *H. juglandinum*, *H. velutinum*, and *L. hongheensis*, as saprophytes from *J. regia*. *D. vulgaris* has been reported in Austria and Thailand (Trouillas et al., 2011; Hyde et al., 2017; Long et al., 2021), and saprobic in decaying twigs of Rutaceae, Oleaceae, Anacardiaceae, and Vitaceae. Diverse species in *Rhytidhysteron* are likely to have jumped hosts from surrounding plants and are unlikely to be a specialist, as *R. subrufulum* has been reported on plants in Rutaceae, Moraceae, Fabaceae, Juglandaceae, and Calycanthaceae. Conversely, *H. juglandinum* appears to be a common species known in *Juglans* and is apparently confined to that host. In addition, the type of species of the genus *H. velutinum* is associated with the dead plant material of the families Adoxaceae, Aquifoliaceae, Asteraceae, Berberidaceae, Betulaceae, Brassicaceae, Cornaceae, Ebenaceae, Fabaceae, Fagaceae, Juglandaceae, Lamiaceae, Magnoliaceae, Menispermaceae, Platanaceae, Poaceae, Polygonaceae, Rosaceae, Tiliaceae, and Ulmaceae, including submerged wood in terrestrial ecosystems (Zhu et al., 2016; Farr and Rossman, 2022). The genus *Loculosulcatispora* was introduced by its anamorphic characteristics by Ren et al. (2020), and the teleomorphic characteristics were first described with the discovery of *L. hongheensis* (Wanasinghe et al., 2022). It comprises two fungi, which are saprobic species in dead wood or twigs of unidentified plants and currently reported in Thailand and China. Our results emphasize that Sichuan province has not yet been properly studied and is an open field for new fungal discoveries. Cheek et al. (2020) proposed that over 50% of plant species have been discovered and that around 90% of fungal species have remained undescribed. As proposed by Hyde (2022), research carried out on various aspects would provide better answers to fungus-host relationships. Hitherto, information on the association of fungi with walnut substrates has been extremely incomplete. An updated checklist of fungi is urgently needed.

## Data availability statement

The data presented in this study are deposited in the NCBI GenBank (accession numbers OP058981–OP059001; OP055918–OP055934; OP055909–OP055916; and OP066330–OP066369), and MycoBank (845074 and 845075).

## Author contributions

C-LY and Y-GL designed the investigation. C-LY and ZZ contributed for the research fund. C-LY, F-HW, CL, B-XW, and H-BY performed the sample collection and handling and the literature study. F-HW and X-LX conducted the preliminary analysis and pathogenicity test. X-LX analyzed the data and wrote the manuscript. C-LY revised and approved the final version of the manuscript. All authors contributed to the article and approved the submitted version.

## Footnotes

1
http://www.MycoBank.org

2
http://blast.ncbi.nlm.nih.gov

## Supplementary material

SUPPLEMENTARY FIGURE 1 Randomized axelerated maximum likelihood (RAxML) tree based on a combined dataset of ITS, *tef*1-a, and *act* sequences in the *Cladosporium cladosporioides* species complex. Out-group taxa are *Cl. longissimum* (CBS 300.96) and *Cl. sphaerospermum* (CBS 193.54). Maximum likelihood (ML) bootstrap support values equal to or above 60% and Bayesian posterior probabilities (PPs) equal to or above 0.94 are shown at the nodes. Isolates from type specimens are in bold. The species characterized in this study are in red. The scale bar represents the expected number of nucleotide substitutions per site.

SUPPLEMENTARY FIGURE 2 RAxML tree based on a combined dataset of ITS and *tub*2 sequences in *Diatrypella* species. Out-group taxon is *Neoeutypella baoshanensi* (GMB0052, LC 12111). Maximum likelihood (ML) bootstrap support values equal to or above 60% and Bayesian posterior probabilities (PPs) equal to or above 0.95 are shown at the nodes. Isolates from type specimens are in bold. The species characterized in this study are in red. The scale bar represents the expected number of nucleotide substitutions per site.

SUPPLEMENTARY FIGURE 3 RAxML tree based on a combined dataset of ITS, LSU, SSU, *rpb*2, and *tef*1-α sequences in *Helminthosporium* species. Out-group taxon is *Massarina cisti* (CBS 266.62). Maximum likelihood (ML) bootstrap support values equal to or above 60% and Bayesian posterior probabilities (PPs) equal to or above 0.95 are shown at the nodes. Isolates from type specimens are in bold. The species characterized in this study are in red. The scale bar represents the expected number of nucleotide substitutions per site.

SUPPLEMENTARY FIGURE 4 RAxML tree based on a combined dataset of ITS, LSU, SSU, *rpb*2, and *tef*1-α sequences in *Sulcatisporaceae* species. Out-group taxa are *L. phraeana* (MFLUCC 18-0472), and *L. aseptata* (MFLUCC 17-2423). Maximum likelihood (ML) bootstrap support values equal to or above 50% and Bayesian posterior probabilities (PPs) equal to or above 0.95 are shown at the nodes. Isolates from type specimens are in bold. The species characterized in this study are in red. The scale bar represents the expected number of nucleotide substitutions per site.

SUPPLEMENTARY FIGURE 5 RAxML tree based on a combined dataset of ITS, *rpb*2, *tef*1-α, and *tub*2 sequences in *Neofusicoccum* species. Out-group taxon is *Botryosphaeria dothidea* (CBS 100564). Maximum likelihood (ML) bootstrap support values equal to or above 60% and Bayesian posterior probabilities (PPs) equal to or above 0.95 are shown at the nodes. Isolates from type specimens are in bold. The species characterized in this study are in red. The scale bar represents the expected number of nucleotide substitutions per site.

SUPPLEMENTARY FIGURE 6 RAxML tree based on a combined dataset of ITS, *ms*204, and *tef*1-α sequences in *Ophiognomonia* species. Out-group taxon is *Ambarignomonia petiolorum* (CBS 121227). Maximum likelihood (ML) bootstrap support values equal to or above 60% and Bayesian posterior probabilities (PPs) equal to or above 0.95 are shown at the nodes. Isolates from type specimens are in bold. The species characterized in this study are in red. The scale bar represents the expected number of nucleotide substitutions per site.

SUPPLEMENTARY FIGURE 7 RAxML tree based on a combined dataset of ITS, LSU, SSU, and *tef*1-α sequences in *Periconia* species. Out-group taxa are *Morosphaeria ramunculicola* (NBRC 107813) and *M. velatispora* (NBRC 107812). Maximum likelihood (ML) bootstrap support values equal to or above 60% and Bayesian posterior probabilities (PPs) equal to or above 0.95 are shown at the nodes. Isolates from type specimens are in bold. The species characterized in this study are in red. The scale bar represents the expected number of nucleotide substitutions per site.

SUPPLEMENTARY FIGURE 8 RAxML tree based on a combined dataset of ITS, LSU, SSU, and *tef*1-α sequences in *Rhytidhysteron* species. Out-group taxon is *Hysterographium fraxini* (MFLU 15-3681, CBS 109.43). Maximum likelihood (ML) bootstrap support values equal to or above 60% and Bayesian posterior probabilities (PPs) equal to or above 0.95 are shown at the nodes. Isolates from type specimens are in bold. The species characterized in this study are in red. The scale bar represents the expected number of nucleotide substitutions per site.

SUPPLEMENTARY FIGURE 9 RAxML tree based on a combined dataset of ITS, LSU, SSU, and *tef*1-α sequences in *Sphaerulina* species. Out-group taxon is *Septoria scabiosicola* (CBS 102334, CBS 108981). Maximum likelihood (ML) bootstrap support values equal to or above 60% and Bayesian posterior probabilities (PPs) equal to or above 0.95 are shown at the nodes. Isolates from type specimens are in bold. The species characterized in this study are in red. The scale bar represents the expected number of nucleotide substitutions per site.

SUPPLEMENTARY TABLE 1 Species, specimens/culture numbers, and GenBank accession numbers of sequences used in this study (newly generated sequences are indicated in bold).
