# Supplementary figures and images for: Morphology and phylogeny of ascomycetes associated with walnut trees (Juglans regia) in Sichuan province, China
Source: Front Microbiol. 2022 Oct 20;13:1016548. doi: 10.3389/fmicb.2022.1016548 (PMC9632355; doi:10.3389/fmicb.2022.1016548)

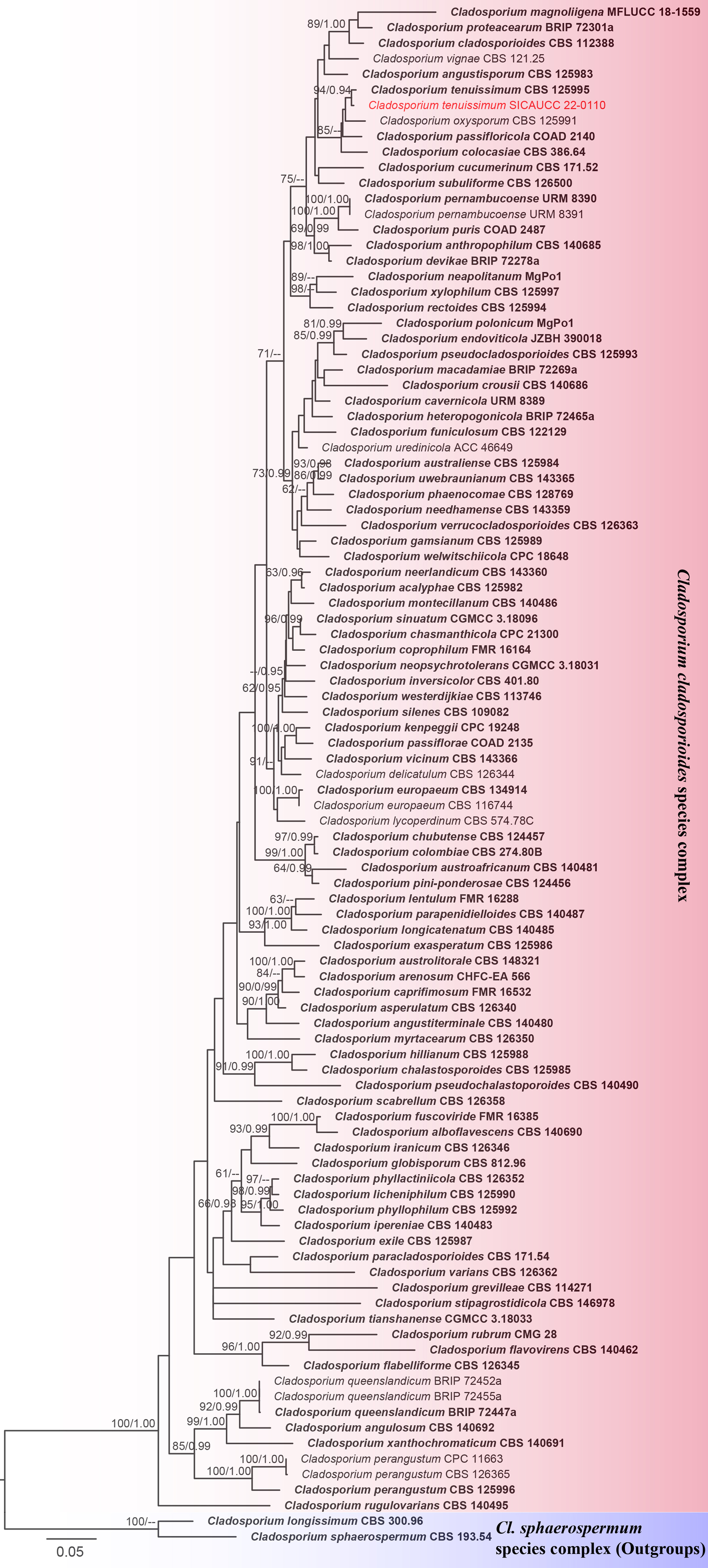

Supplement: SUPPLEMENTARY FIGURE 1 — Randomized axelerated maximum likelihood (RAxML) tree based on a combined dataset of ITS, tef1-a, and act sequences in the Cladosporium cladosporioides species complex. Out-group taxa are Cl. longissimum (CBS 300.96) and Cl. sphaerospermum (CBS 193.54). Maximum likelihood (ML) bootstrap support values equal to or above 60% and Bayesian posterior probabilities (PPs) equal to or above 0.94 are shown at the nodes. Isolates from type specimens are in bold. The species characterized in this study are in red. The scale bar represents the expected number of nucleotide substitutions per site. [file Image_1.JPEG]

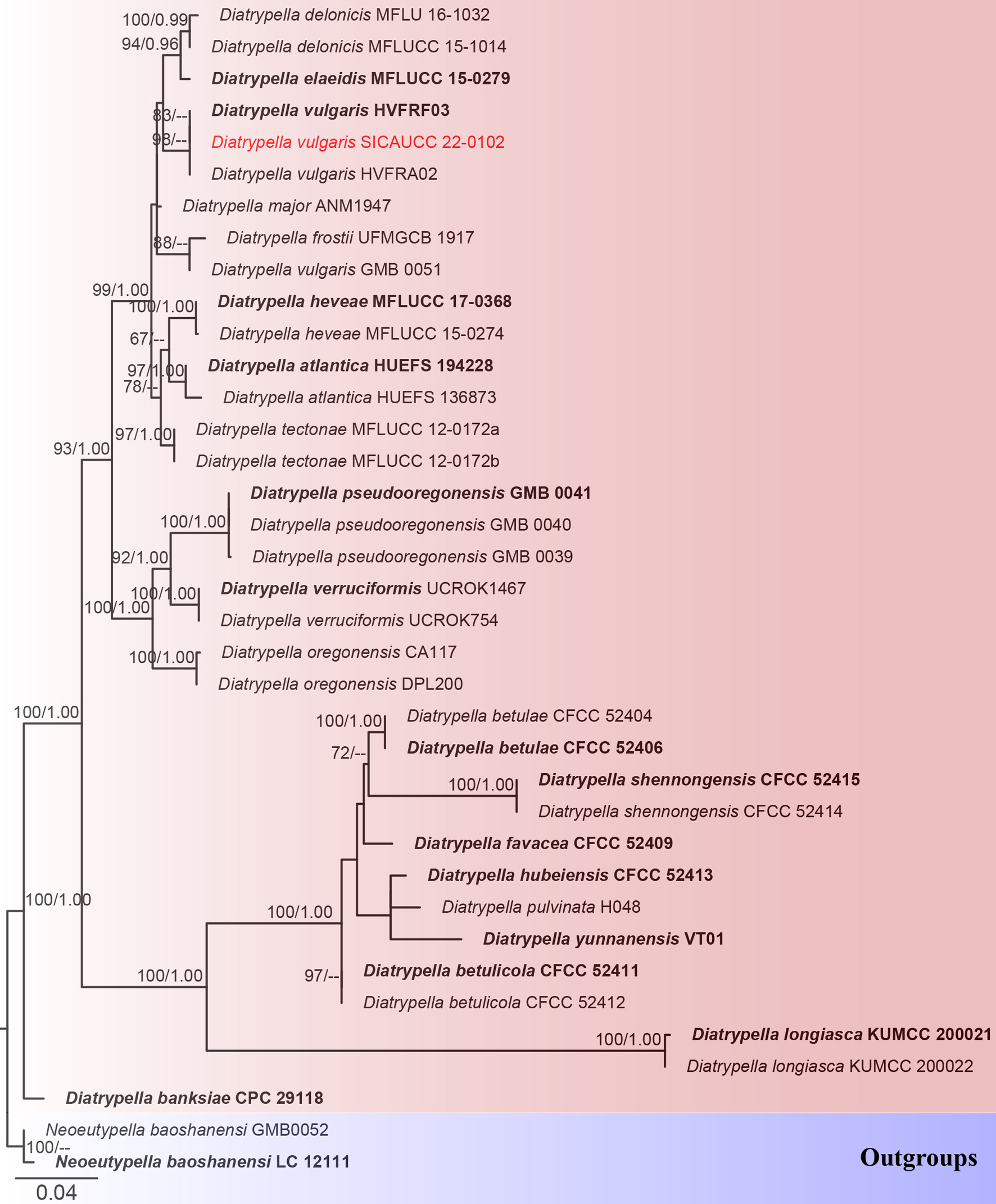

Supplement: SUPPLEMENTARY FIGURE 2 — RAxML tree based on a combined dataset of ITS and tub2 sequences in Diatrypella species. Out-group taxon is Neoeutypella baoshanensi (GMB0052, LC 12111). Maximum likelihood (ML) bootstrap support values equal to or above 60% and Bayesian posterior probabilities (PPs) equal to or above 0.95 are shown at the nodes. Isolates from type specimens are in bold. The species characterized in this study are in red. The scale bar represents the expected number of nucleotide substitutions per site. [file Image_2.JPEG]

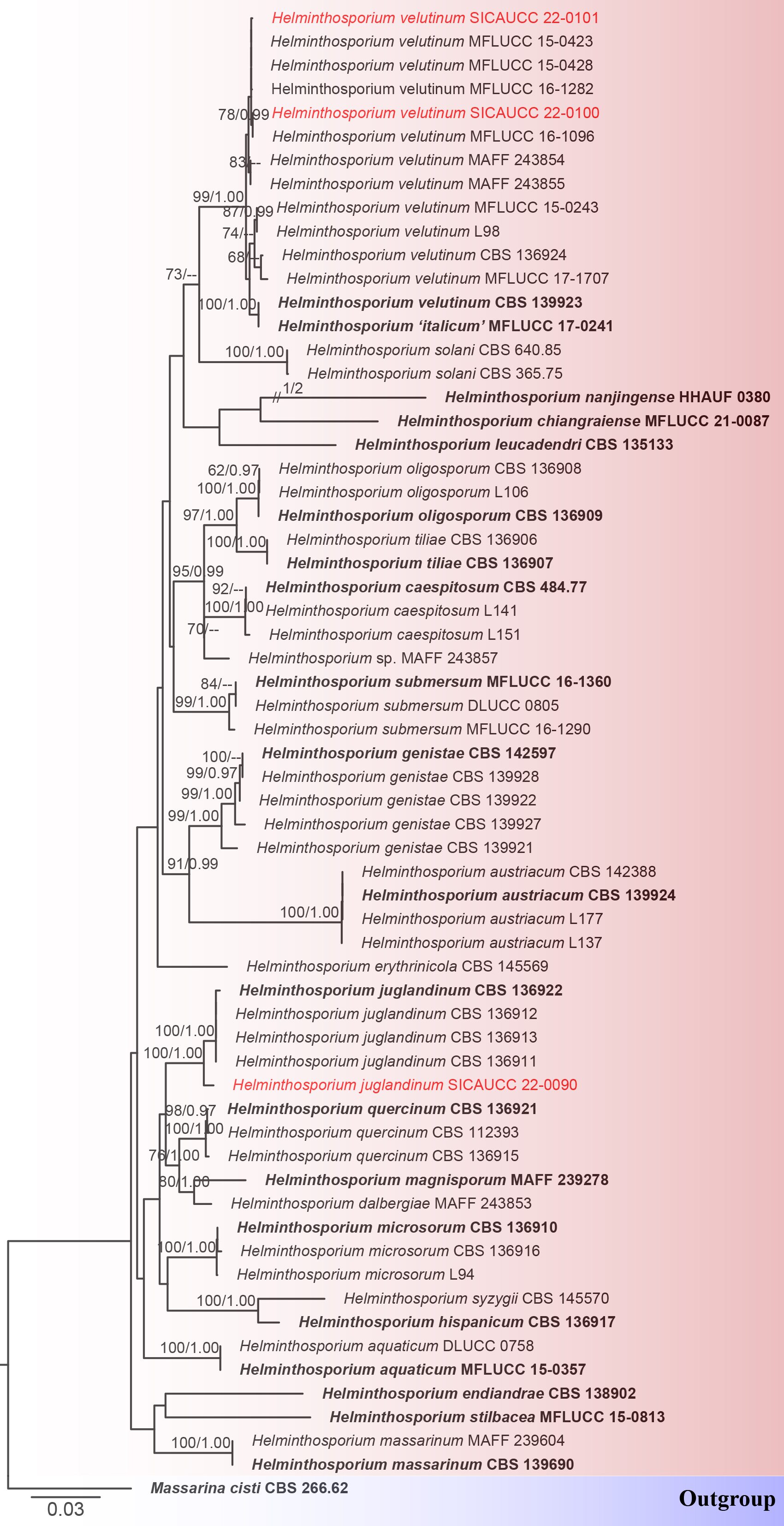

Supplement: SUPPLEMENTARY FIGURE 3 — RAxML tree based on a combined dataset of ITS, LSU, SSU, rpb2, and tef1-α sequences in Helminthosporium species. Out-group taxon is Massarina cisti (CBS 266.62). Maximum likelihood (ML) bootstrap support values equal to or above 60% and Bayesian posterior probabilities (PPs) equal to or above 0.95 are shown at the nodes. Isolates from type specimens are in bold. The species characterized in this study are in red. The scale bar represents the expected number of nucleotide substitutions per site. [file Image_3.JPEG]

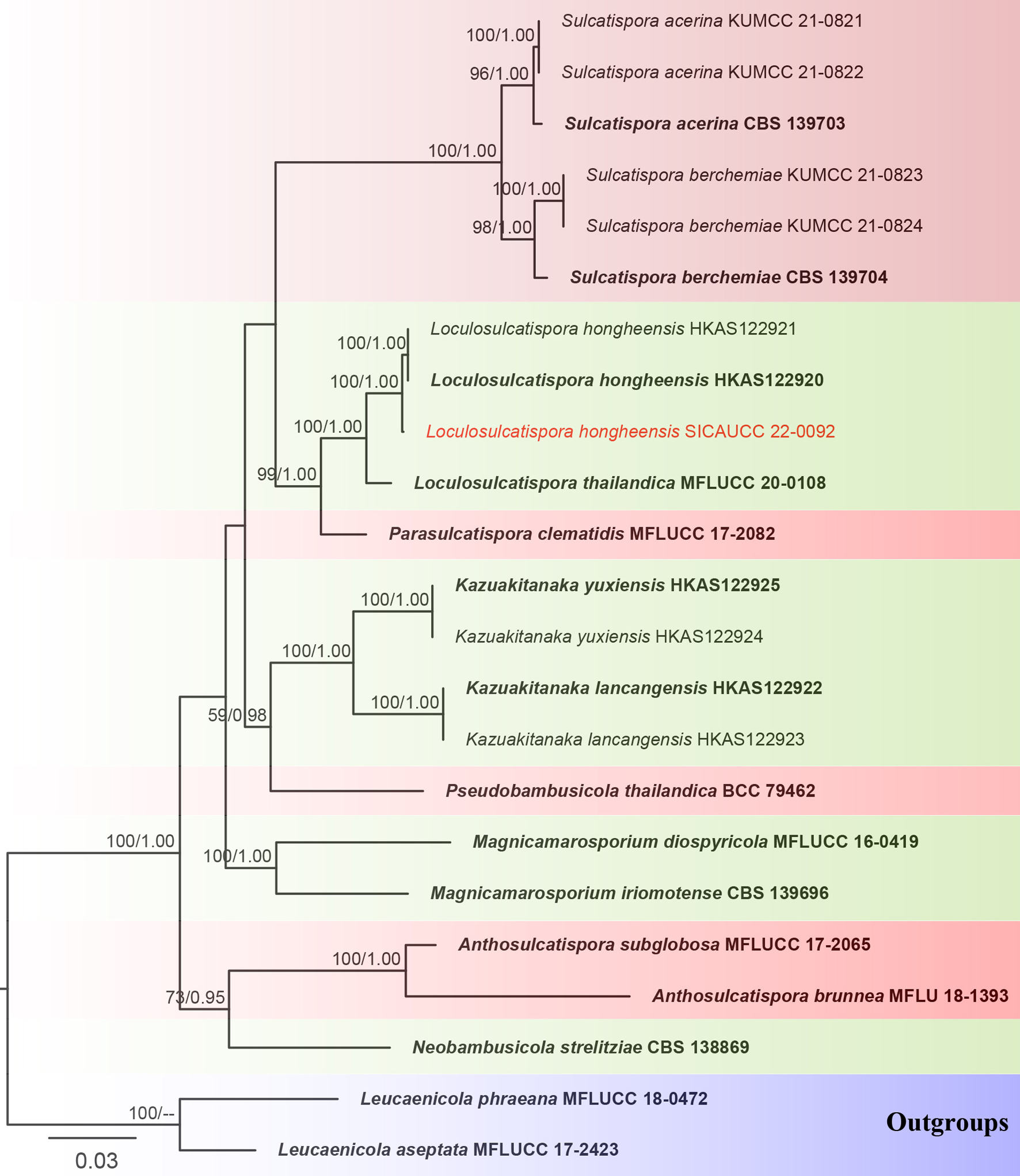

Supplement: SUPPLEMENTARY FIGURE 4 — RAxML tree based on a combined dataset of ITS, LSU, SSU, rpb2, and tef1-α sequences in Sulcatisporaceae species. Out-group taxa are L. phraeana (MFLUCC 18-0472), and L. aseptata (MFLUCC 17-2423). Maximum likelihood (ML) bootstrap support values equal to or above 50% and Bayesian posterior probabilities (PPs) equal to or above 0.95 are shown at the nodes. Isolates from type specimens are in bold. The species characterized in this study are in red. The scale bar represents the expected number of nucleotide substitutions per site. [file Image_4.JPEG]

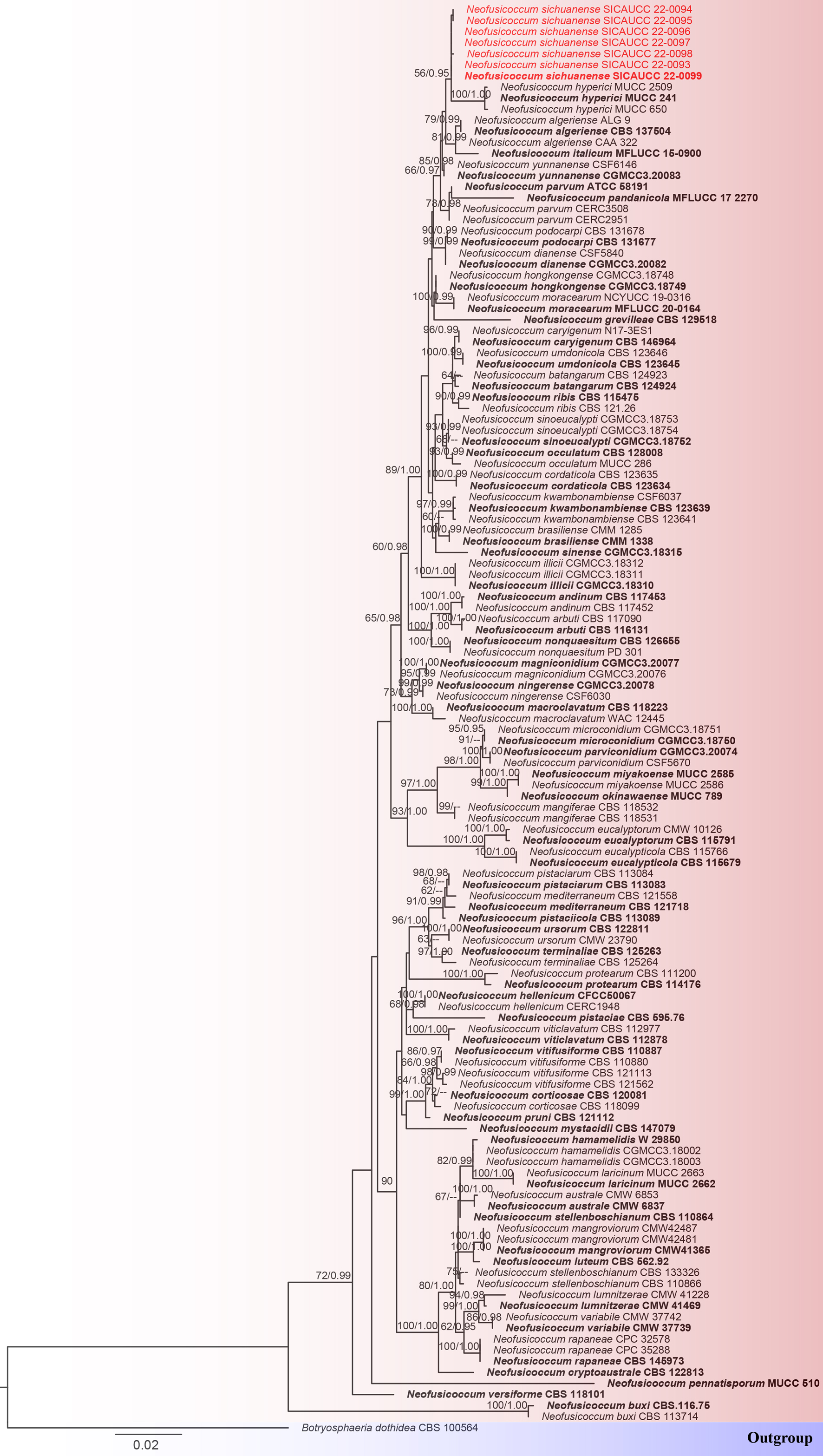

Supplement: SUPPLEMENTARY FIGURE 5 — RAxML tree based on a combined dataset of ITS, rpb2, tef1-α, and tub2 sequences in Neofusicoccum species. Out-group taxon is Botryosphaeria dothidea (CBS 100564). Maximum likelihood (ML) bootstrap support values equal to or above 60% and Bayesian posterior probabilities (PPs) equal to or above 0.95 are shown at the nodes. Isolates from type specimens are in bold. The species characterized in this study are in red. The scale bar represents the expected number of nucleotide substitutions per site. [file Image_5.JPEG]

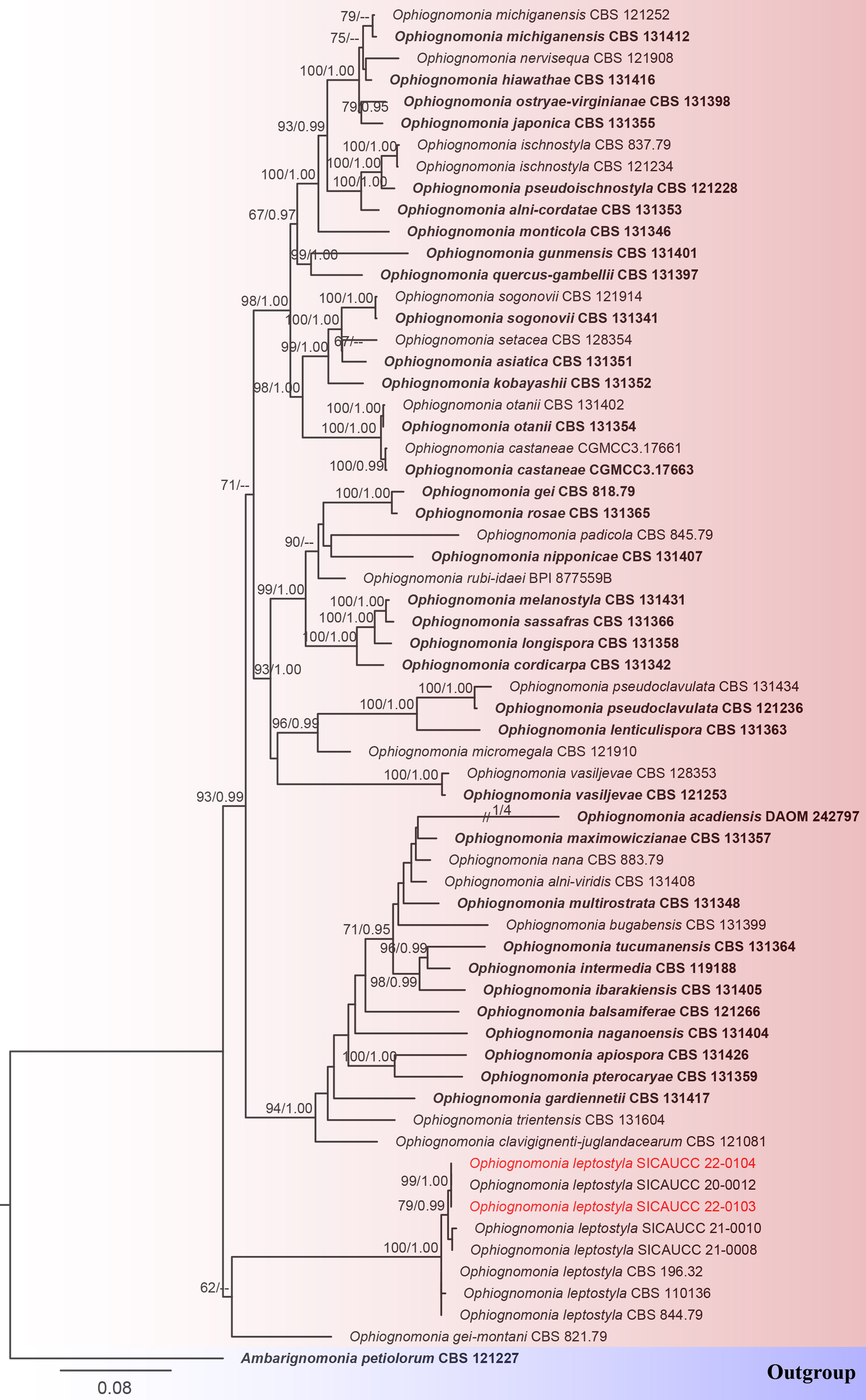

Supplement: SUPPLEMENTARY FIGURE 6 — RAxML tree based on a combined dataset of ITS, ms204, and tef1-α sequences in Ophiognomonia species. Out-group taxon is Ambarignomonia petiolorum (CBS 121227). Maximum likelihood (ML) bootstrap support values equal to or above 60% and Bayesian posterior probabilities (PPs) equal to or above 0.95 are shown at the nodes. Isolates from type specimens are in bold. The species characterized in this study are in red. The scale bar represents the expected number of nucleotide substitutions per site. [file Image_6.JPEG]

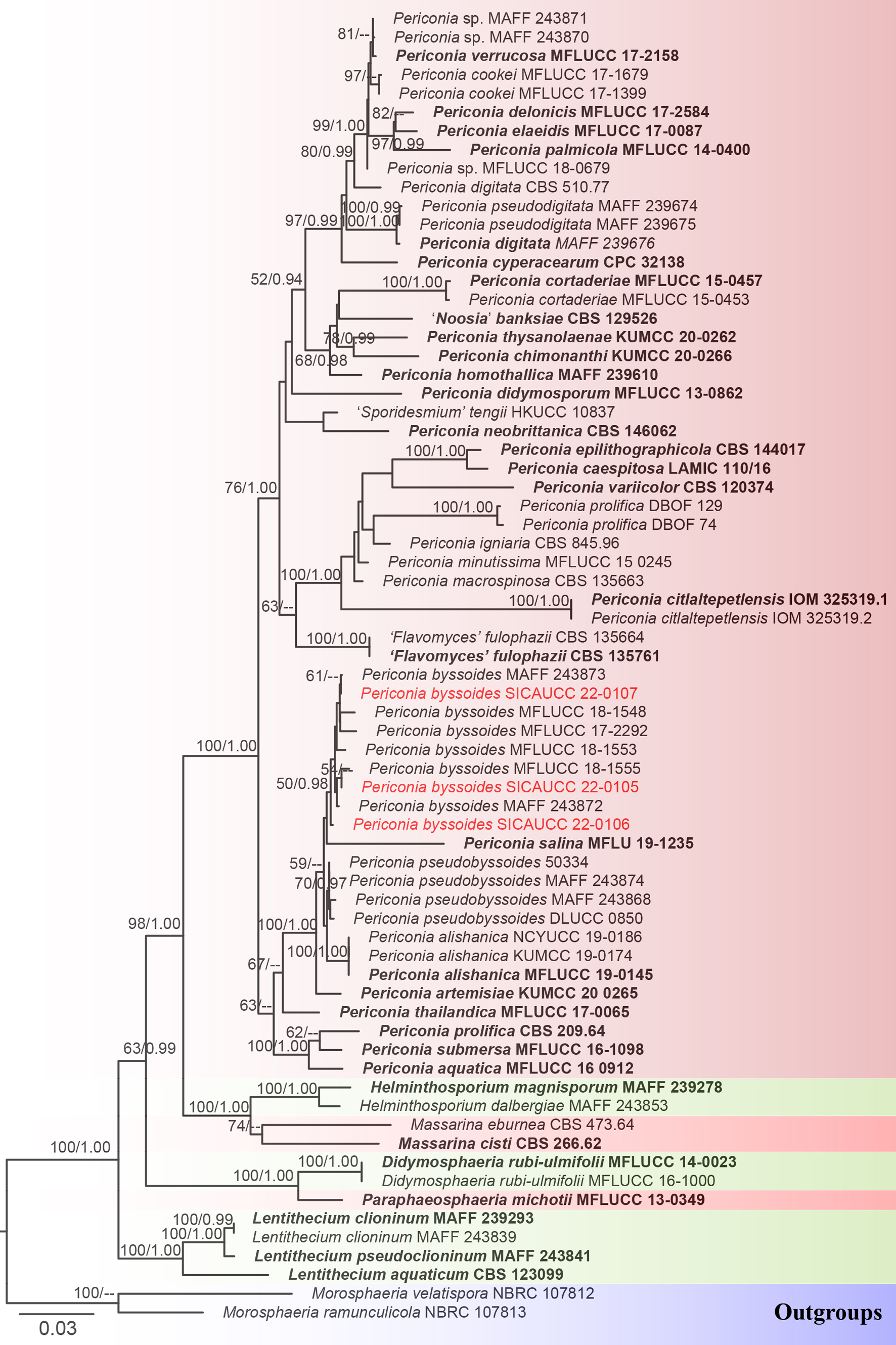

Supplement: SUPPLEMENTARY FIGURE 7 — RAxML tree based on a combined dataset of ITS, LSU, SSU, and tef1-α sequences in Periconia species. Out-group taxa are Morosphaeria ramunculicola (NBRC 107813) and M. velatispora (NBRC 107812). Maximum likelihood (ML) bootstrap support values equal to or above 60% and Bayesian posterior probabilities (PPs) equal to or above 0.95 are shown at the nodes. Isolates from type specimens are in bold. The species characterized in this study are in red. The scale bar represents the expected number of nucleotide substitutions per site. [file Image_7.JPEG]

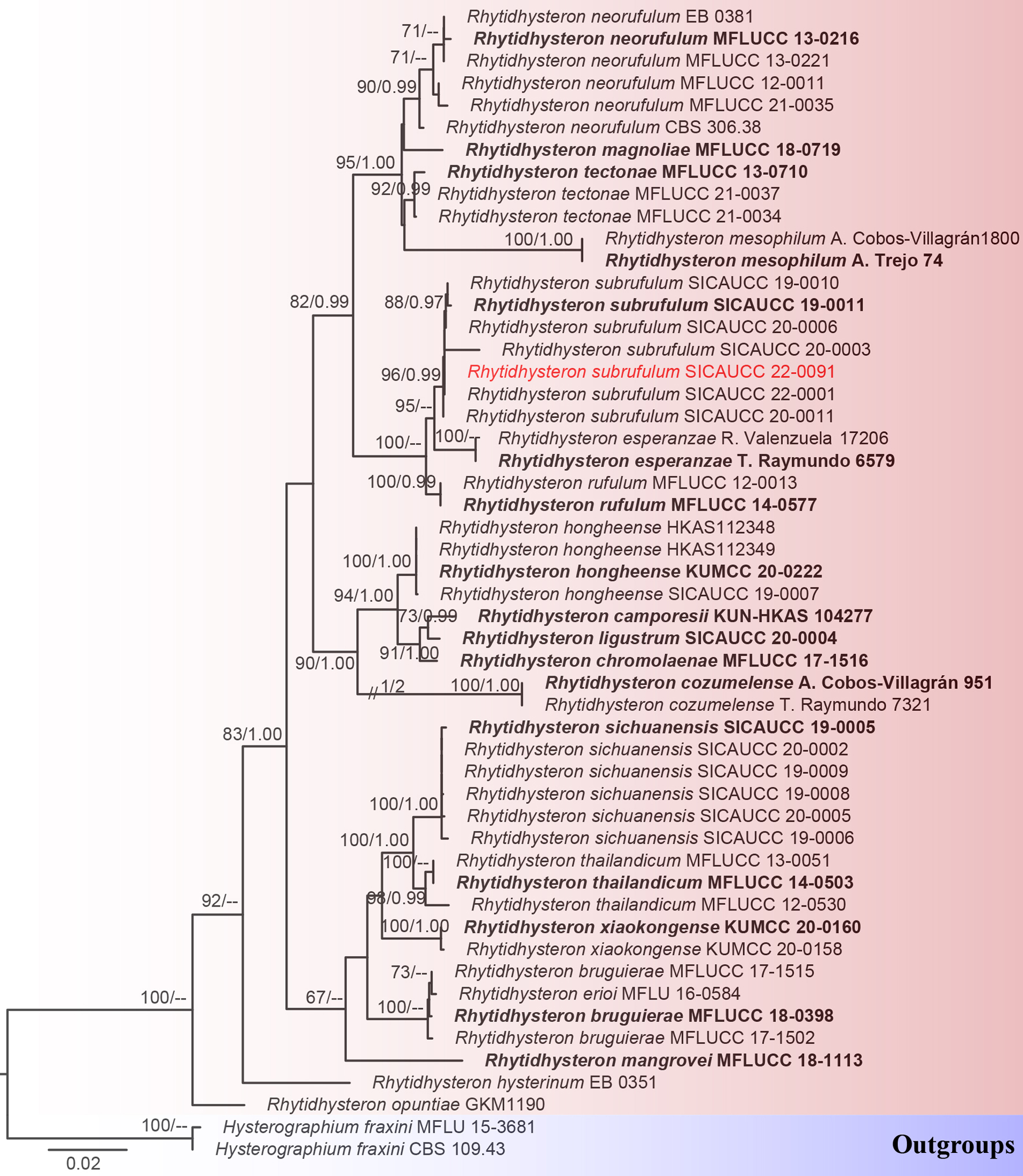

Supplement: SUPPLEMENTARY FIGURE 8 — RAxML tree based on a combined dataset of ITS, LSU, SSU, and tef1-α sequences in Rhytidhysteron species. Out-group taxon is Hysterographium fraxini (MFLU 15-3681, CBS 109.43). Maximum likelihood (ML) bootstrap support values equal to or above 60% and Bayesian posterior probabilities (PPs) equal to or above 0.95 are shown at the nodes. Isolates from type specimens are in bold. The species characterized in this study are in red. The scale bar represents the expected number of nucleotide substitutions per site. [file Image_8.JPEG]

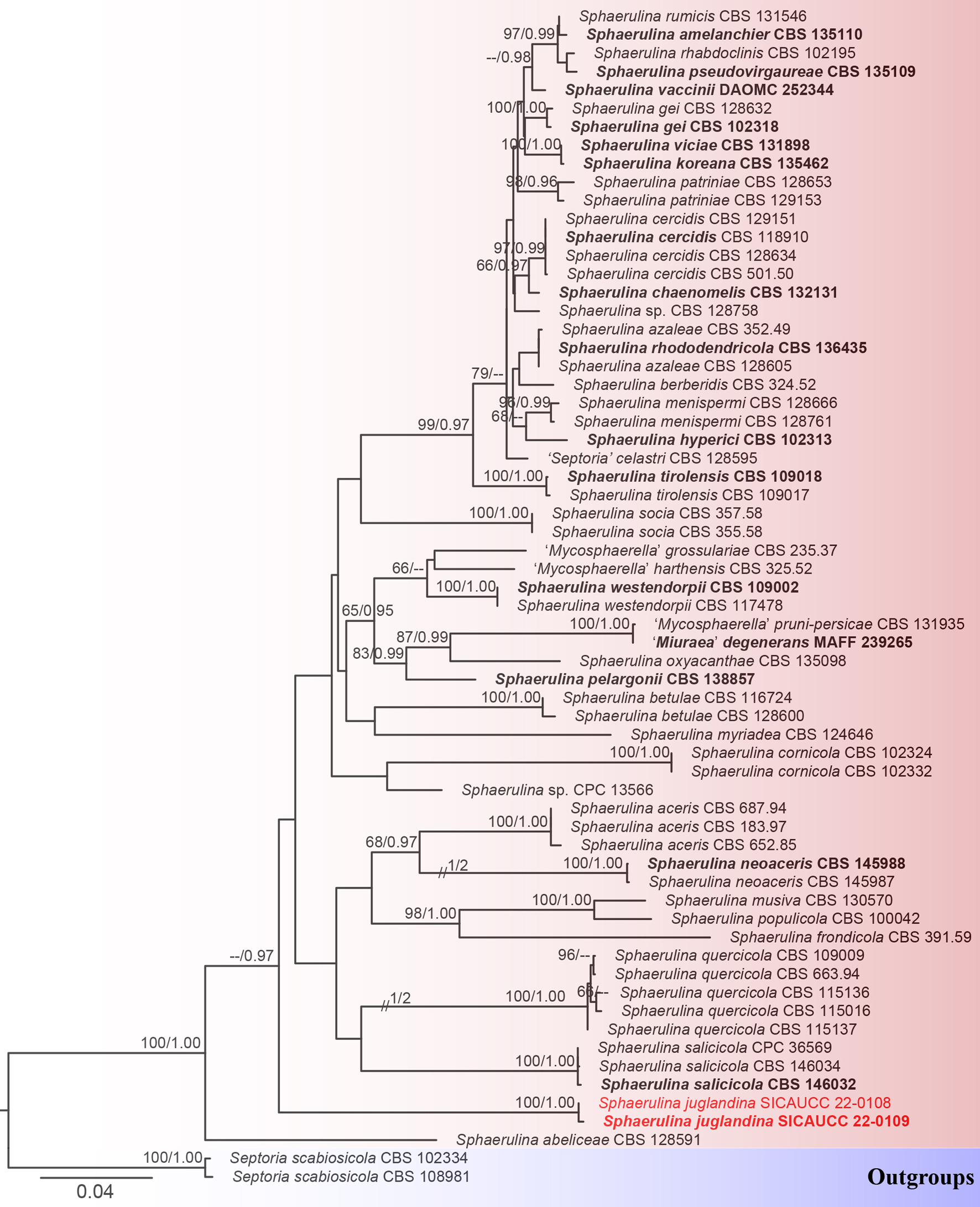

Supplement: SUPPLEMENTARY FIGURE 9 — RAxML tree based on a combined dataset of ITS, LSU, SSU, and tef1-α sequences in Sphaerulina species. Out-group taxon is Septoria scabiosicola (CBS 102334, CBS 108981). Maximum likelihood (ML) bootstrap support values equal to or above 60% and Bayesian posterior probabilities (PPs) equal to or above 0.95 are shown at the nodes. Isolates from type specimens are in bold. The species characterized in this study are in red. The scale bar represents the expected number of nucleotide substitutions per site. [file Image_9.JPEG]
